# Minibrain/Dyrk1a Regulates Food Intake through the Sir2-FOXO-sNPF/NPY Pathway in *Drosophila* and Mammals

**DOI:** 10.1371/journal.pgen.1002857

**Published:** 2012-08-02

**Authors:** Seung-Hyun Hong, Kyu-Sun Lee, Su-Jin Kwak, Ae-Kyeong Kim, Hua Bai, Min-Su Jung, O-Yu Kwon, Woo-Joo Song, Marc Tatar, Kweon Yu

**Affiliations:** 1 Aging Research Centre, Korea Research Institute of Bioscience and Biotechnology (KRIBB), Daejeon, Korea; 2 Functional Genomics Program, University of Science and Technology (UST), Daejeon, Korea; 3 Department of Ecology and Evolutionary Biology, Brown University, Providence, Rhode Island, United States of America; 4 Institute for Brain Science and Technology, FIRST Research Group, Inje University, Busan, Korea; 5 Department of Anatomy, School of Medicine, Chungnam National University, Daejeon, Korea; Buck Institute, United States of America

## Abstract

Feeding behavior is one of the most essential activities in animals, which is tightly regulated by neuroendocrine factors. *Drosophila melanogaster* short neuropeptide F (sNPF) and the mammalian functional homolog neuropeptide Y (NPY) regulate food intake. Understanding the molecular mechanism of sNPF and NPY signaling is critical to elucidate feeding regulation. Here, we found that *minibrain* (*mnb*) and the mammalian ortholog *Dyrk1a* target genes of sNPF and NPY signaling and regulate food intake in *Drosophila melanogaster* and mice. In *Drosophila melanogaster* neuronal cells and mouse hypothalamic cells, sNPF and NPY modulated the *mnb* and *Dyrk1a* expression through the PKA-CREB pathway. Increased Dyrk1a activated Sirt1 to regulate the deacetylation of FOXO, which potentiated FOXO-induced *sNPF/NPY* expression and in turn promoted food intake. Conversely, AKT-mediated insulin signaling suppressed FOXO-mediated *sNPF/NPY* expression, which resulted in decreasing food intake. Furthermore, human *Dyrk1a* transgenic mice exhibited decreased FOXO acetylation and increased *NPY* expression in the hypothalamus, as well as increased food intake. Our findings demonstrate that Mnb/Dyrk1a regulates food intake through the evolutionary conserved Sir2-FOXO-sNPF/NPY pathway in *Drosophila melanogaster* and mammals.

## Author Summary

Feeding behavior is one of the most essential activities in animals. Abnormal feeding behaviors cause metabolic syndromes including obesity and diabetes. Neuropeptides regulate feeding behavior in animals from nematode to human. Here, we presented molecular genetic evidences of how neuropeptides regulate food intake using fruit fly and mouse model systems. *Drosophila* short neuropetide F (sNPF) and the mammalian functional homolog neuropeptide Y (NPY) are produced from neurons in the brain of fruit fly and mouse, respectively. These neuropeptides turned on the *minibrain*, in mammals also called *Dyrk1a*, a target gene through the PKA-CREB pathway. Then, this Mnb/Dyrk1a enzyme activated Sir2/Sirt1 enzyme, which activated FOXO transcriptional factor, turning on the expression of a *sNPF/NPY* target gene. The increased sNPF/NPY increased food intake in fruit flies and mice. On the contrary, increased food intake induced insulin and activated insulin signaling. When insulin signaling is activated, FOXO transcriptional factor inhibited expression of a *sNPF/NPY* target gene. The inhibited sNPF/NPY reduced food intake. These findings indicate that FOXO transcription factor acts as a gatekeeper for fasting–feeding transition by regulating *sNPF/NPY* expression in *Drosophila* and mammals.

## Introduction

Neuropeptides regulate a wide range of physiological processes in animals. In mammals, NPY is widely distributed in the brain and involved in various physiological functions including food intake. In the mammalian brain, the hypothalamus is the center for controlling food intake. The hypothalamic injection of NPY in the rat brain induces hyperphagia and obesity. In the hypothalamus, the arcuate nucleus (ARC) that contains orexigenic NPY and AgRP expressing neurons and anorexigenic POMC neurons senses hormonal levels of insulin and leptin and regulates food intake [1]. In *Drosophila*, sNPF, a functional homolog of NPY produced in sNPFnergic neurons of the fly brain, regulates food intake and growth [2]. Recently, we reported that sNPF and sNPF receptor (sNPFR1) regulate body growth through evolutionary conserved ERK-mediated insulin signaling in *Drosophila* and rat insulinoma cells [3].

*Drosophila* Minibrain (Mnb) and its mammalian ortholog Dual specificity tyrosine-phosphorylation-regulated kinase 1a (Dyrk1a) are highly expressed in the neural tissues [4], [5], [6]. The *Dyrk1a* gene has been implicated in Down Syndrome (DS) [5], [7] and the expression level of *Dyrk1a* is increased in DS patients and Ts65Dn mice, a mouse model of Down syndrome [4], [8]. Mutations of *mnb* and *Dyrk1a* in *Drosophila* and mammals show neural phenotypes like defects in neuroblasts proliferation and brain development [6], [9]. Human patients with truncated mutations in the *Dyrk1a* gene also show microcephaly [10], [11]. To date, however, the effects of *mnb* and *Dyrk1a* upon food intake have not been described.

FoxO1 modulates food intake by regulation of orexigenic *Argp* and anorexigenic *Pomc* genes in the hypothalamus of mice. In the ARC of hypothalamic neurons, FoxO1 is localized in the nuclei during fasting and in the cytoplasm by feeding [12]. Sirtuin1 (Sirt1), the mammalian ortholog of *Drosophila* Silent information regulator 2 (Sir2), in the ARC also regulates food intake [13]. The Sirt1 protein level increases during fasting. Sirt1 inhibition by the hypothalamic knock-out in the AgRP neurons decreases food intake [14]. In N43 hypothalamic cells, pharmacological inhibition of Sirt1 increases anorexigenic *POMC* expression but co-treatment with Sirt1 inhibitor and FoxO1 siRNA does not [15], suggesting that Sirt1-mediated FoxO1 deactylation is involved in the regulation of *POMC* mRNA and food intake.

In this study, we identified *mnb* and *Dyrk1a* as target genes of sNPF and NPY signaling, respectively, and describe a molecular mechanism of how Mnb and Dyrk1a regulate food intake in *Drosophila* and mice.

## Results

### sNPF Targets *mnb* to Regulate Food Intake in *Drosophila*

**Figure 1:**
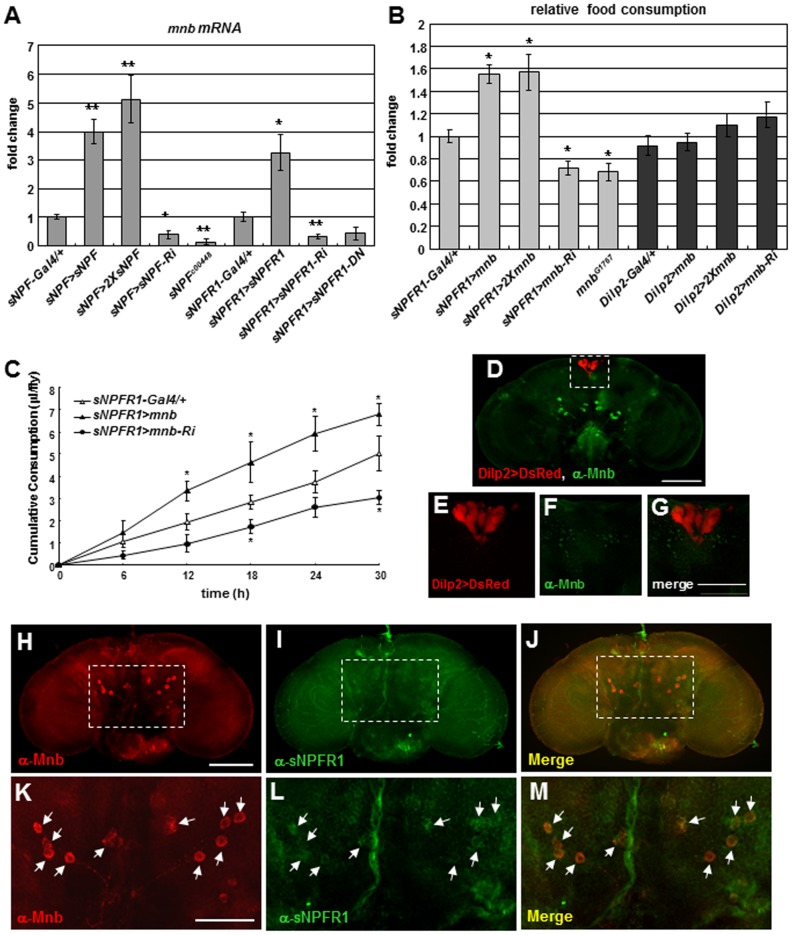
Expression and distribution of *Drosophila mnb* in adults in relation to *sNPF*, *sNPFR1*, and feeding. (A) *mnb* mRNA prepared from fly heads was measured by RT-qPCR. *mnb* mRNA was increased relative to *sNPF-Gal4* and *sNPFR1-Gal4* controls when *sNPF* and *sNPFR1* was overexpressed in sNPFnergic neurons (*sNPF>sNPF, sNPF>2XsNPF*) and in sNPFR1 neurons (*sNPFR1>sNPFR1*). *mnb* mRNA was decreased when *sNPF* and *sNPFR1* were inhibited (*sNPF>sNPF-Ri, sNPF^c00448^, sNPFR1>sNPFR1-Ri, sNPFR1>sNPFR1-DN*). (B) Food consumption measured by the colormetric assay. Relative to *sNPFR1-Gal4* control, *mnb* overexpression in sNPFR1 neurons (*sNPFR1>mnb, sNPFR1>2Xmnb*) increased feeding whereas *mnb* suppression (*sNPFR1>mnb-Ri, mnb^G1767^*) decreased feeding. Overexpression or inhibition of *mnb* in the insulin producing cells with the *Dilp2-Gal4* driver (*Dilp2>mnb, Dilp2>2Xmnb, Dilp2>mnb-Ri*) did not change the feeding. (C) Food consumption measured by CAFÉ assay. Relative to the *sNPFR1-Gal4* (open triangle) control, *sNPFR1>mnb* (closed triangle) increased while *sNPFR1>mnb-Ri* (closed circle) decreased cumulative food consumption. Data are presented as means ± s.e.m. from three independent experiments. *P<0.05, **P<0.001 (One-way ANOVA analysis). (D-G) Neurons of the *Drosophila* adult brain expressing Mnb protein (green) do not overlap with insulin producing cells marked with *Dilp2>DsRed* (red). (H-M) Mnb protein expression neurons (H, K, red) and sNPFR1 protein expression neurons (I, L, green) were overlapped in the median neurons (J, dot box; M, arrows). Scale bars are 100 µm (D, H) and 50 µm (G, K).

To find genes affected by sNPF signaling, we performed a DNA microarray analysis using the Affymetrix *Drosophila* Genome 2.0 Array GeneChip with mRNA extracted from *Drosophila* neuronal BG2-c6 cells treated with sNPF peptide. Among the 159 genes with at least a two-fold change, mRNA of *mnb* increased 34-fold compared to the control (Table S1). To test whether the expression of *mnb* is dependent on sNPF signaling *in vivo*, we examined the expression levels of *mnb* in *sNPF* and *sNPFR1* mutants. When *sNPF* was overexpressed in sNPFnergic neurons with the *sNPF-Gal4* driver [16] (*sNPF>sNPF, sNPF>2XsNPF*), *mnb* mRNA increased 4 to 5-fold compared with the *sNPF-Gal4*. mRNA of *mnb* decreased by less than half when *sNPF* was inhibited (*sNPF>sNPF-Ri*) or by an *sNPF* mutant (*sNPF^c00448^*) (Figure 1A and Figure S1A). When *sNPFR1* was overexpressed via a *sNPFR1-Gal4* driver (Figure S2) (*sNPFR1>sNPFR1*), *mnb* mRNA was increased 3-fold compared with the *sNPFR1-Gal4* control. When *sNPFR1* was inhibited (*sNPFR1>sNPFR1-Ri*) or suppressed (*sNPFR1>sNPFR1-DN*), *mnb* mRNA was decreased by more than 50% (Figure 1A and Figure S1A). Like *mnb* mRNA, Mnb proteins were also increased in *sNPF* or *sNPFR1* overexpression with the *sNPF-Gal4* or *sNPFR1-Gal4* driver, (*sNPF>2XsNPF*, *sNPFR1>sNPFR1*) while reduced in an *sNPF* mutant (*sNPF^c00448^*) or *sNPFR1* inhibition (*sNPFR1>sNPFR1-Ri*) compared with the *sNPF-Gal4* or *sNPFR1-Gal4* control (Figure S3A). However, the numbers of Mnb expression neurons (asterisks) are consistent in the *sNPFR1-Gal4* control, *sNPFR1* overexpression (*sNPFR1>sNPFR1*), *sNPFR1* inhibition (*sNPFR1>sNPFR1-Ri*), and an *sNPF^c00448^* mutant (Figure S3B–S3F). These results indicate that sNPF-sNPFR1 signaling regulates *mnb* mRNA and protein expression in *Drosophila*.

To understand how Mnb protein may interact with the sNPFR1 receptor, we immunostained fly adult brains with Mnb and sNPFR1 antibodies. The Mnb antibody produced strong and weak staining in neuronal cells (Figure 1H, 1K, red) while the sNPFR1 receptor antibody stained many neurons (Figure 1I, 1L, green). Among the strongly stained Mnb neurons, cell bodies of symmetrically localized median neurons behind the antennal lobe show overlap with the antibody against sNPFR1 (Figure 1J, 1M, arrows). At least ten neuronal cell bodies in median neurons were stained with the both antibodies. This coincidence suggests that at least part of Mnb function may be regulated by sNPF-sNPFR1 signaling.

Since sNPF signaling regulates food intake and growth, and growth is regulated by ERK-mediated insulin signaling [3], we hypothesized that sNPF may regulate food intake through the *mnb* gene. To assess this hypothesis, we used the CAFÉ assay [17] to measure feeding in *mnb* mutant adults. Because homozygous *mnb* deletion mutants (*mnb^d305^* and *mnb^d419^*) generated by the imprecise excisions of the P-element (Figure S4A) are lethal (as are homozygous *Dyrk1a* mutant mice) we analyzed *mnb* overexpression and hypomorphs generated by RNAi. *mnb* overexpression in sNPFR1 neurons (*sNPFR1>mnb*) increased cumulative food consumption compared to the *sNPFR1-Gal4* control whereas inhibiting *mnb* (*sNPFR1>mnb-Ri*) decreased cumulative food consumption (Figure 1C), indicating that *mnb* expression in sNPFR1 neurons can regulate food intake. Likewise, we measured the amount of food intake by the amount of digested dye from colored food. Overexpression of *mnb* in sNPFR1 neurons (*sNPFR1>mnb* and *sNPFR1>2Xmnb*) increased consumed dye up to 57% compared with that of the *sNPFR1-Gal4* control whereas *mnb* inhibition (*sNPFR1>mnb-Ri*) or the *mnb* mutant (*mnb^G1767^*) decreased this intake by 30% (Figure 1B and Figure S1B). As expected, levels of *mnb* mRNA and protein were markedly reduced by *mnb* inhibition and by the *mnb^G1767^* mutant relative to the *sNPFR1-Gal4* and *w-* controls (Figure S4B, S4C). Since sNPFR1 signaling in the insulin producing cells (IPCs) regulates body growth through insulin signaling [3], we examined the effect of *mnb* in IPCs upon food intake. However, food intake was not affected by *mnb* overexpression in IPCs driven via *Dilp2-Gal4* (*Dilp2>mnb* and *Dilp2>2Xmnb*) or by *mnb* inhibition in IPCs (*Dilp2>mnb-Ri*) (Figure 1B). Expression of *mnb* in sNPFR1 neurons but not in IPCs (Figure 1D–1G) is sufficient to regulate food intake.

To determine the consequences of *mnb* control upon food intake we measured the body weight of young adults from mutant and control. Overexpression of *mnb* in sNPFR1 neurons (*sNPFR1>mnb*) increased body weight relative to that of *sNPFR1-Gal4* controls, similar to the effect seen when *sNPFR1* is overexpressed (*sNPFR1>sNPFR1*). On the contrary, body weight is decreased when *mnb* is repressed in sNPFR1 neurons (*sNPFR1>mnb-Ri*) and *mnb^G1767^* mutant (Figure S4D). The amounts of food intake in the mutants were similar when they were normalized to body mass or to the number of flies (Figure S4E).

Since *mnb* is involved in neural development [6], [9], we restricted *mnb* expression in the adult stage using the *tub-GAL80ts* inducible system [18] and tested food intake. *mnb* overexpression (*sNPFR1-Gal4+tubGal80ts>mnb, sNPFR1-Gal4+tubGal80ts>2Xmnb*) and *mnb* inhibition (*sNPFR1-Gal4+tubGal80ts>mnb-Ri*) flies were cultured in the 22°C permissive temperature until adulthood to suppress *sNPFR1-Gal4* expression by the *tubGal80ts*. Then, these adult flies were shifted to the 30°C restrictive temperature in which the *tubGal80ts* cannot suppress *sNPFR1-Gal4*. In the permissive condition, the *mnb* overexpression and *mnb* inhibition flies did not change the amount of food intake compared with the control flies (*sNPFR1-Gal4; tub-Gal80ts*) (Figure S5A). However, in the restrictive condition, the *mnb* overexpression increased food intake compared with the control and the *mnb* inhibition suppressed food intake (Figure S5B). These results indicate that the food intake phenotype of *mnb* mutants is not due to developmental effects.

### sNPF-PKA-CREB-*mnb* Signaling in *Drosophila* Neuronal BG2-c6 Cells

**Figure 2:**
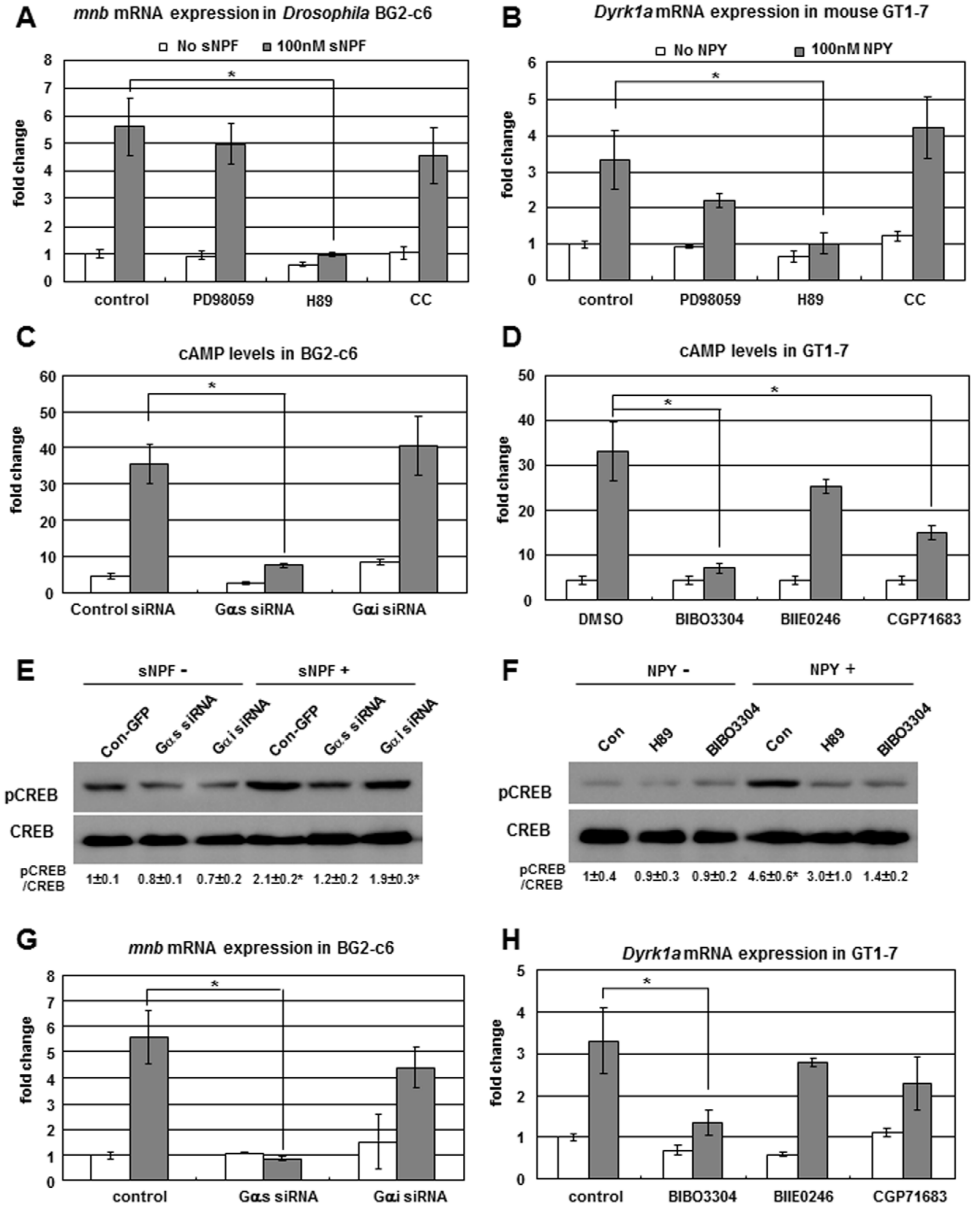
sNPF/NPY-sNPFR1/NPYR1-PKA-CREB-mnb/Dyrk1a signaling in *Drosophila* neuronal BG2-c6 cells and mouse hypothalamic GT1-7 cells. (A) *mnb* mRNA in *Drosophila* neuronal BG2-c6 cells increased in response to treatment with sNPF peptide, but not when co-treated with H89 PKA inhibitor. The ERK inhibitor PD98059 and PKC inhibitor CC did not suppress sNPF-induced *mnb* expression. (B) *Dyrk1a* mRNA in mouse hypothalamic GT1-7 cells increased in response to treatment with NPY peptide, but not when co-treated with the PKA inhibitor H89. (C) In *Drosophila* BG2-c6 cells, sNPF peptide induced cAMP, while transfection of cells with *Gαs* siRNA but not *Gαi* siRNA repressed this effect. (D) In mouse GT1-7 cells, NPY peptide induced cAMP, while co-treatment with NPYR1 inhibitor BIBO3304 but not NPYR2 and NPYR5 inhibitors strongly decreased this effect. (E) Western blot to detect activated CREB (pCREB) in *Drosophila* BG2-c6 cells. sNPF peptide increasd pCREB but not in cells transfected with *Gαs* siRNA. (F) Western blot to detect activated CREB (pCREB) in mouse GT1-7 cells. NPY peptide increased pCREB but not when cells are co-treated with PKA inhibitor H89 or NPYR1 inhibitor BIBO3304. (G) In *Drosophila* BG2-c6 cells, sNPF peptide induced *mnb* mRNA, while transfection of cells with *Gαs* siRNA but not *Gαi* siRNA repressed this effect. (H) In mouse GT1-7 cells, NPY peptide induced *Dyrk1a* mRNA, while co-treatment with NPYR1 inhibitor BIBO3304 but not NPYR2 and NPYR5 inhibitors strongly decreased this effect. Data are presented as means ± s.e.m. from three independent experiments. *P<0.05 (One-way ANOVA analysis).

To study how sNPFR1 regulates *mnb* expression, we treated *Drosophila* central nervous system-derived BG2-c6 cells [19] with synthetic sNPF peptide, which changed *sNPF* and *sNPFR1* expression slightly (Figure S6A). Consistent with our initial observations and with patterns in genetically manipulated flies, sNPF treatment increased *mnb* mRNA more than 5-fold compared to the control when measured by quantitative PCR (Figure 2A). Then, we tested whether the induction of this *mnb* mRNA is mediated by ERK, as we have previously observed for the induction of *Drosophila* insulin like peptides (*Dilps*) by sNPF [3]. However, ERK inhibitor PD98059 treatment of the sNPF peptide-treated cells did not suppress the *mnb* expression. On the other hand, sNPFR1 is a G-protein coupled receptor (GPCR), and the second messenger of GPCRs is cAMP or Ca^++^ which respectively activates PKA or PKC [20].Thus, we treated BG2-c6 cells with the protein kinase A (PKA) inhibitor H89 or with protein kinase C (PKC) inhibitor Chelerythrine Chloride (CC). H89 decreased both basal and sNPF-induced *mnb* expression level but the PKC inhibitor CC showed no effect (Figure 2A). sNPF signaling appears to control *mnb* expression through PKA, not through ERK or PKC. Consistent with this interpretation, BG2-c6 cells treated with sNPF showed increased levels of cAMP in a time-dependent manner, peaking at 15 min (Figure S6B).

To find the Gα subunit of the sNPFR1 G-protein heterotrimer, we examined Gαs and Gαi, both of which modulate cAMP [20]. When transfected into BG2-c6 cells Gαs siRNA inhibited sNPF-induced cAMP whereas transfection with Gαi siRNA did not (Figure 2C), suggesting that Gαs is a Gα subunit of sNPFR1 that can modulate the cAMP-PKA pathway in *Drosophila* neuronal cells. Next, we examined the activation of the cAMP responding element binding protein (CREB), which is a PKA down-stream transcription factor [21]. sNPF stimulated the phosphorylation of CREB in control cells whereas Gαs siRNA transfection suppressed this sNPF dependent activation of CREB (Figure 2E). In addition, Gαs siRNA transfection completely blocked the induction of *mnb* by sNPF, but Gαi siRNA transfection did not (Figure 2G). These data indicate that Gαs is a key Gα subunit of the sNPFR1 G-protein as it regulates *mnb* expression. Taken together, these findings demonstrate that sNPF signaling effectively regulates *mnb* expression through the Gαs-cAMP-PKA-CREB pathway in *Drosophila* neuronal cells.

### NPY-PKA-CREB-*DYRK1A* Signaling in Mouse Hypothalamic GT1-7 Cells

To compare the functional conservation of sNPF-sNPFR1-PKA-CREB-*mnb* signaling with the signaling of mammalian NPY, we conducted similar experiments with mouse GT1-7 hypothalamic cells [22]. NPY treatment increased *Dyrk1a* mRNA while the PKA inhibitor H89 strongly suppressed NPY-induced *Dyrk1a* expression (Figure 2B). NPY signaling activates *Dyrk1a* expression through PKA, much like the PKA mediated *mnb* expression by sNPF in fly neuronal cells. Next, we measured the cAMP level in the NPY treated GT1-7 cells. As expected, cAMP level increased time-dependently and peaked at 15 min (Figure S6C). Five NPY receptors (NPYR1, 2, 4, 5, and 6) mediate the NPY signal [23]. Among them, NPYR1, 2, and 5 receptors are broadly expressed in the mouse nervous system and mediate NPY-induced food intake [24]. We treated GT1-7 cells with chemical inhibitors against these receptors: BIBO3304 for NPYR1, BIIE0246 for NPYR2, and CGP71683 for NPYR5. The NPYR1 inhibitor BIBO3304 substantially decreased the NPY-induced cAMP level; little effect was seen for the inhibitors of NPYR2 and NPYR5 (Figure 2D). Thus, NPY appears to activate the cAMP-PKA pathway mainly through NPYR1 in GT1-7 hypothalamic cells. Next, we measured the CREB activation. As expected, inhibiting PKA or NPYR1 suppressed the NPY-induced activation of CREB (Figure 2F), confirming that NPY signal is mediated through NPYR1-cAMP-PKA-CREB. In addition, the NPYR1 inhibitor strongly suppressed NPY-induced *Dyrk1a* expression; this was not seen with the inhibitors of NPYR2 and NPYR5 (Figure 2H). Taken together, these findings indicate that NPY signaling regulates *Dyrk1a* expression mainly through the NPYR1-cAMP-PKA-CREB pathway in mouse hypothalamic cells. Importantly, this signal transduction pathway is conserved between fly neuronal cells and mammalian hypothalamic cells.

### Genetic Interactions among *sNPFR1*, *Gαs*, *PKA*, *CREB*, and *mnb* Genes, and CREB ChIP Analysis

**Figure 3:**
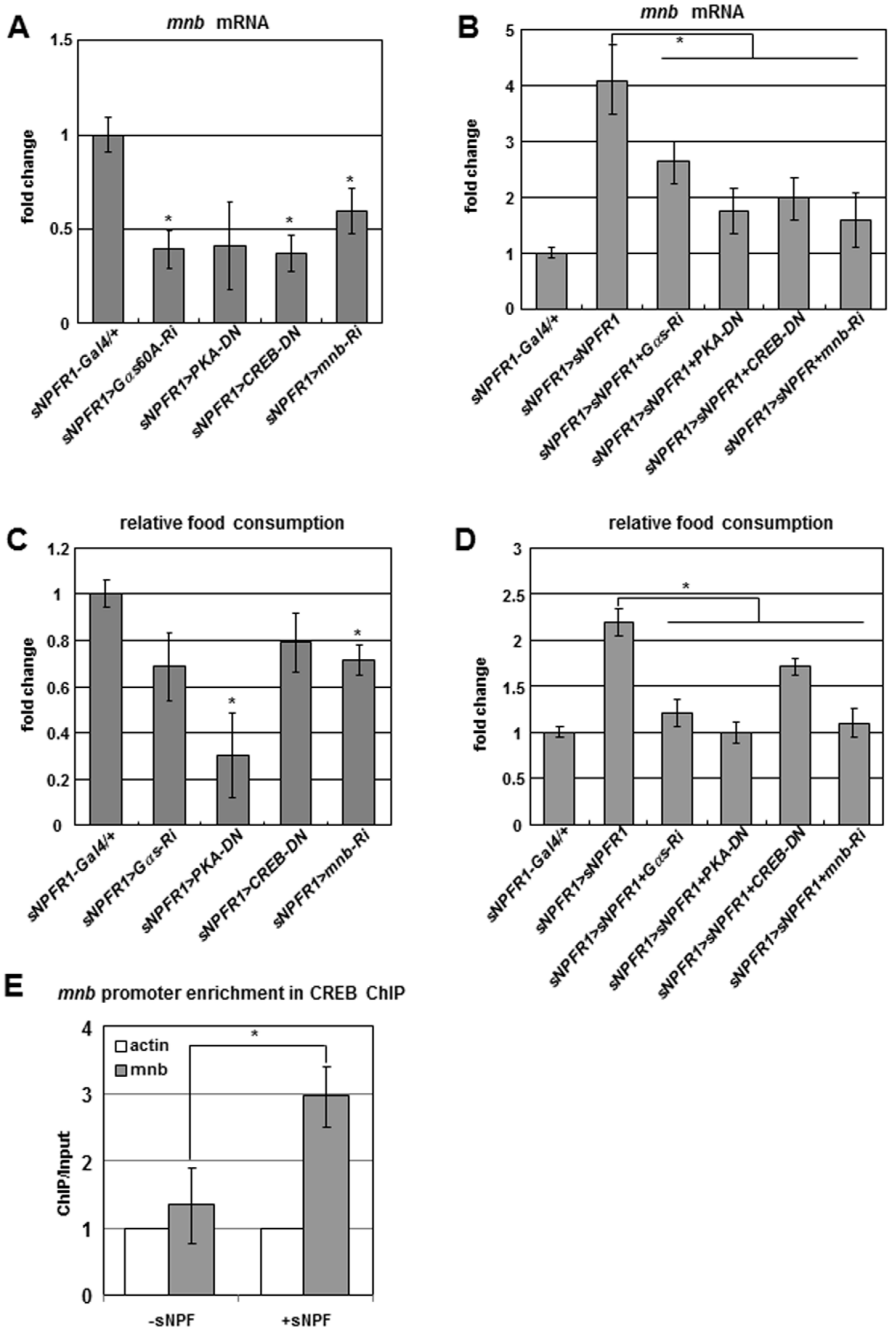
Genetic interactions among *sNPFR1*, *Gαs*, *PKA*, *CREB*, and *mnb* genes and CREB ChIP–PCR analysis. (A, C) *mnb* mRNA (A) and feeding (C) were reduced by suppressing *Gαs, PKA, CREB, and mnb* in sNPFR1 neurons relative to *sNPFR1-Gal4* control. (B, D) *mnb* mRNA (B) and feeding (D) were reduced by suppressing *Gαs*, *PKA*, *CREB*, and *mnb* while overexpressing *sNPFR1* in sNPFR1 neurons relative to overexpressing *sNPFR1* alone (*sNPFR1>sNPFR1*). (E) In *Drosophila* BG2-c6 cells. CREB binding was enriched at the promoter region of the *mnb* gene by 3-fold compared to the *Act5c* and sNPF peptide non-treated controls (ChIP-PCR). Data are presented as means ± s.e.m. from three independent experiments. *P<0.05 (One-way ANOVA analysis).

To study genetic interactions among *sNPFR1*, *Gαs*, *PKA*, *CREB*, and *mnb* genes, we suppressed *Gαs*, *PKA*, *CREB*, and *mnb* by RNAi and Dominant Negative (DN) forms in neurons that simultaneously overexpressed *sNPFR1*. Each of these suppression genotypes reduced the level of *mnb* mRNA compared with *sNPFR1-Gal4* and *UAS* controls (Figure 3A and Figure S7A). In contrast to the strong induction of *mnb* produced by *sNPFR1* overexpression alone (*sNPFR1>sNPFR1*), *mnb* induction was inhibited in genotypes where *sNPFR1* overexpression occurred with each of the suppression constructs (*sNPFR1>sNPFR1*+*Gαs-Ri*, *sNPFR1>sNPFR1*+*PKA-DN*, *sNPFR1>sNPFR1*+*CREB-DN*, *sNPFR1>sNPFR1*+*mnb-Ri*) (Figure 3B). In sNPFR1 neurons of flies, as in isolated cells, *Gαs*, *PKA*, and *CREB* may work downstream of *sNPFR1* to regulate *mnb* expression. The consequences of these interactions are also seen in terms of food intake. *Gαs*, *PKA*, *CREB*, and *mnb* suppression mutant flies have reduced food intake compared to those of the *sNPFR1-Gal4* and *UAS* controls (Figure 3C and Figures S1C, S7B). Furthermore, increased food intake of *sNPFR1* overexpression was suppressed by co-inhibition of *Gαs*, *PKA*, and *CREB*, respectively (Figure 3D). These results suggest that the sNPFR1 may regulate food intake through *Gαs*, *PKA*, *CREB*, and *mnb*.

Based on promoter analysis of *mnb* genes from twelve *Drosophila* species, we found a conserved cAMP responding element (CRE) site (Figure S8). Interestingly, the promoters of human *Dyrk1a* and mouse *Dyrk1a* genes contain CRE [25]. To test whether CREB binds to the promoter of the *mnb* gene, we performed the chromatin immunoprecipitation (ChIP)-PCR analysis with the CREB antibody in sNPF treated *Drosophila* neuronal BG2-c6 cells. CREB binding was enriched at the sNPF treated promoter region of the *mnb* gene by 3-fold compared to the *Act5C* and sNPF non-treated controls (Figure 3E). Together these *in vivo* and *in vitro* findings indicate that sNPF-sNPFR1-Gαs-PKA-CREB pathway controls expression of the *mnb* target gene and regulates food intake in *Drosophila*.

### Positive Regulation of *sNPF/NPY* by the Mnb/Dyrk1a-Sir2-FOXO Pathway

**Figure 4:**
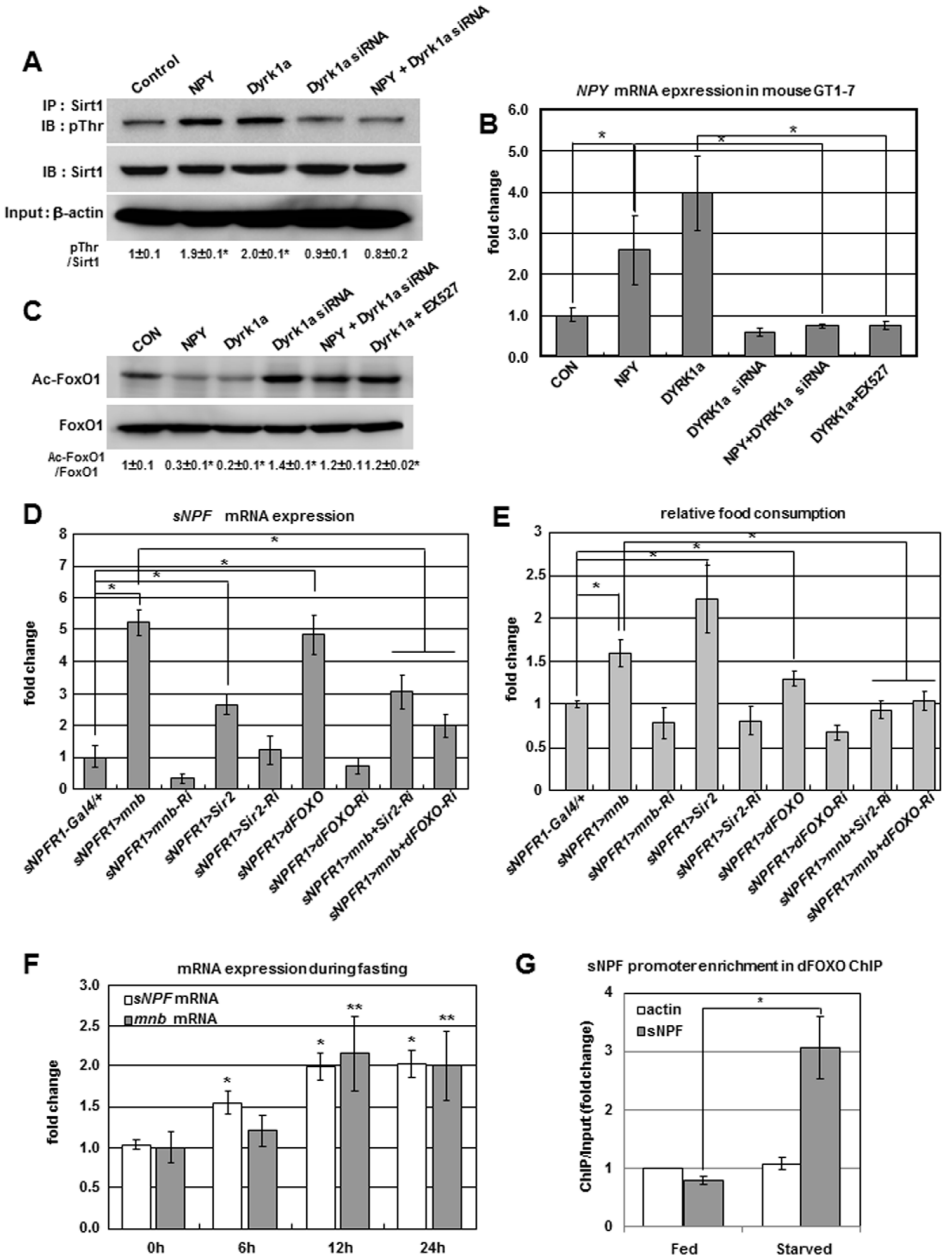
Positive regulation of *sNPF/NPY* by the Mnb/Dyrk1a-Sir2-FOXO pathway. (A) Sirt1 phosphorylation was increased in mouse GT1-7 cells transfected with *Dyrk1a* or treated with NPY but reduced in cells transfected with *Dyrk1a* siRNA or *Dyrk1a* siRNA co-treated with NPY. (B) *NPY* mRNA was increased in GT1-7 cells transfected with *Dyrk1a* or treated with NPY peptide, but reduced in cells transfected with *Dyrk1a* siRNA, *Dyrk1a* siRNA co-treated with NPY, or *Dyrk1a* co-treated with Sirt1 inhibitor EX527. (C) FoxO1 acetylation was reduced in GT1-7 cells transfected with *Dyrk1a* or treated with NPY peptide, but FoxO1 acetylation was increased in cells transfected with *Dyrk1a* siRNA, *Dyrk1a* siRNA co-treated with NPY, or *Dyrk1a* co-treated with Sirt1 inhibitor EX527. (D, E) *sNPF* mRNA (D) and food intake (E) were reduced when *Sir2* or *dFOXO* were inhibited while *mnb* was overexpressed in *sNPFR1* expressing neurons relative to levels observed for *mnb* overexpression alone (*sNPFR1>mnb*). (F) *sNPF* and *mnb* mRNA increased in adults starved 12 h. (G) dFOXO binding to the promoter region of the *sNPF* gene in adult flies starved 12 h was elevated relative to the *Act5c* and fed controls (ChIP-chip). Data are presented as means ± s.e.m. from three independent experiments. *P<0.05, **P<0.001 (One-way ANOVA analysis).

A possible avenue through which Mnb regulates food intake could involve Sirt1/Sir2. Notably, Dyrk1a kinase phosphorylates Sirt1 in HEK293T cells [26], and activated Sirt1 deacetylates FoxO1 to modulate the activity of this transcription factor in the rat hypothalamus [15]. Accordingly we determined if these interactions were present and associated in mouse hypothalamic GT1-7 cells. In cells transfected with *Dyrk1a* or treated with NPY, phosphorylation of Sirt1 was increased as detected by immunoprecipitation with Sirt1 antibody, followed by immunobloting with phospho-threonine (pThr) antibody. Sirt1 phosphorylation was reduced by *Dyrk1a* siRNA or *Dyrk1a* siRNA with NPY (Figure 4A). In addition, FoxO1 acetylation was reduced in cells transfected by *Dyrk1a* or treated with NPY, while FoxO1 acetylation was increased by *Dyrk1a* siRNA, *Dyrk1a* siRNA with NPY, or *Dyrk1a* transfection coupled with the Sirt1 inhibitor EX527 (Figure 4C). Importantly, *NPY* mRNA itself was increased in cells transfected with *Dyrk1a* or treated with NPY peptide, and *NPY* mRNA was decreased by *Dyrk1a* siRNA, *Dyrk1a* siRNA with NPY, or *Dyrk1a* overexpression in the presence of Sirt1 inhibitor (Figure 4B). In mouse hypothalamic GT1-7 cells, Dyrk1a phosphorylates Sirt1 and this activated Sirt1 appears to deacetylate FoxO1 which in turn positively regulates expression of *NPY*.

To study genetic interactions among *mnb*, *Sir2*, and *dFOXO* in an animal model, we manipulated *Sir2* and *dFOXO* in the *Drosophila mnb* overexpression genotype. When *mnb*, *Sir2*, and *dFOXO* were overexpressed in *sNPFR1-Gal4* neurons (*sNPFR1>mnb*, *sNPFR1>Sir2*, *sNPFR1>dFOXO*) (Figure S9A), *sNPF* mRNA and food intake were increased compared to *sNPFR1-Gal4* and *UAS* controls (Figure 4D and 4E, Figure S7B and S7C). Conversely, when *mnb*, *Sir2*, and *dFOXO* were inhibited in *sNPFR1* expressing neurons (*sNPFR1>mnb-Ri*, *sNPFR1>Sir2-Ri*, *sNPFR1>dFOXO-Ri*) (Figure S9B), the expression levels of *sNPF* and food intake were decreased or similar to those of *sNPFR1-Gal4* and *UAS* controls (Figure 4D and 4E, Figure S7B and S7C). Finally the level of *sNPF* mRNA and food intake were reduced in adults when *Sir2* or *dFOXO* were inhibited in sNPFR1 neurons that overexpressed *mnb* (*sNPFR1>mnb+Sir2-Ri, sNPFR1>mnb+dFOXO-Ri*) compared with flies only overexpressing *mnb* (*sNPFR1>mnb*). These data suggest that *mnb* may regulate *sNPF* expression and food intake through *Sir2* and *dFOXO*.

Since fasting can stimulate food intake, we tested whether an acute period of food deprivation affected the expression of *mnb* and *sNPF* of adult flies. Levels of *mnb* and *sNPF* mRNA increased 2-fold after 12 h starvation (Figure 4F). We propose that dFOXO contributes to this expression of *sNPF* in starved flies. We identified a common dFOXO consensus binding site (RWWAACA) in the *sNPF* promoter from twelve *Drosophila* species (Figure S10) and performed a chromatin immunoprecipitation (ChIP)-tiled gene array analysis with dFOXO antibody in fed and starved adult flies. dFOXO binding was enriched at the promoter region of *sNPF* gene more than 3-fold in the starved flies compared to the *Act5c* and fed controls (Figure 4G). These results suggest that the dFOXO transcriptional factor regulates *sNPF* mRNA expression by direct binding to its promoter in *Drosophila*, as seen for FoxO1 regulation of *NPY* expression in mice [27].

Overall, these results from mouse hypothalamic GT1-7 cells and *Drosophila* indicate that the Mnb/Dyrk1a-Sir2-FOXO pathway positively regulates *sNPF/NPY* expression and food intake.

### Negative Regulation of *sNPF/NPY* by Insulin Signaling

**Figure 5:**
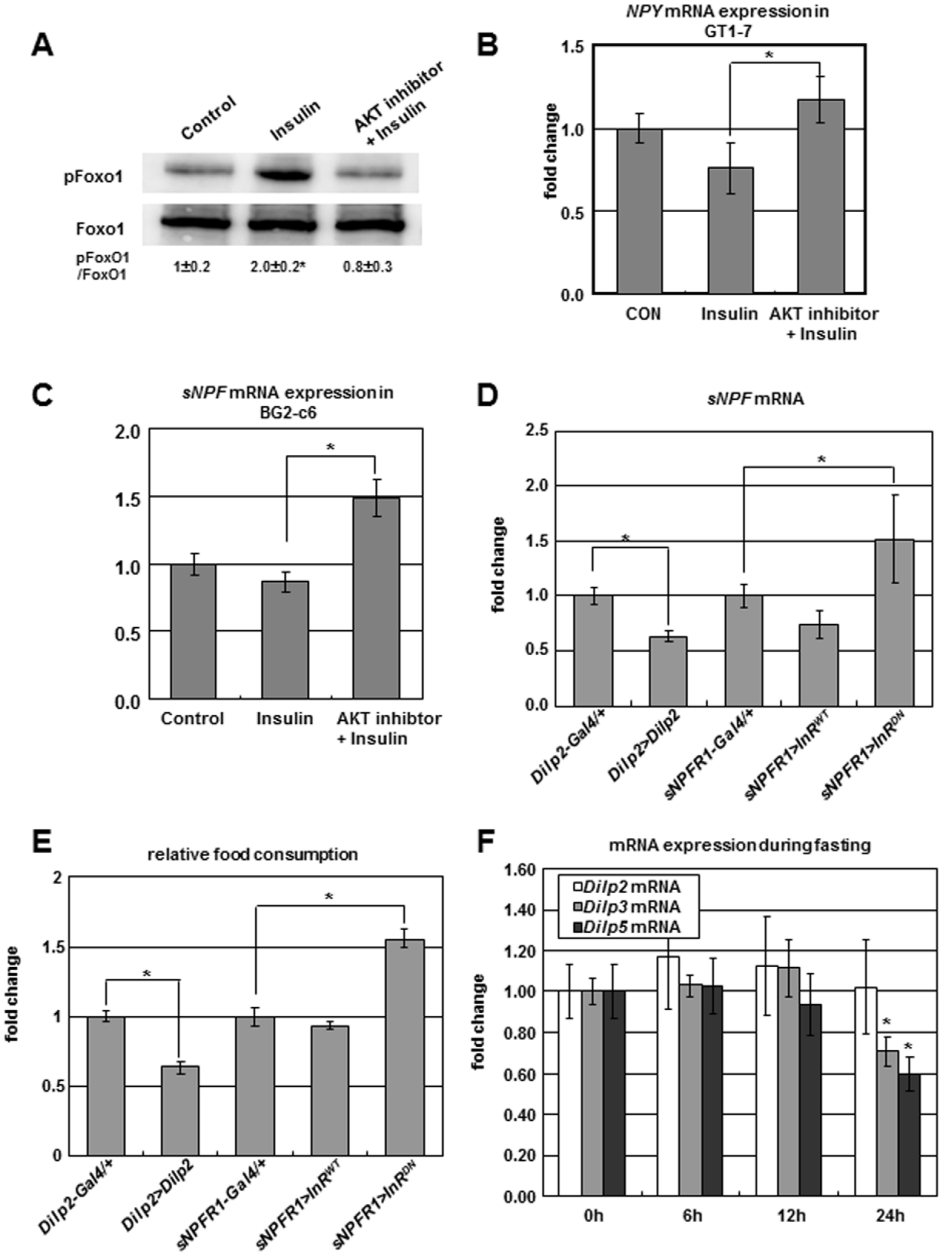
Negative regulation of *sNPF/NPY* by insulin signaling. (A, B) Insulin treatment increased FoxO1 phosphorylation and decreased *NPY* expression in mouse hypothalamic GT1-7 cells while insulin with AKT inhibitor co-treatment slightly decreased FoxO1 phosphorylation and increased *NPY* expression. (C) Insulin with AKT inhibitor co-treatment increased *sNPF* expression in fly neuronal BG2-c6 cells. (D, E) *sNPF* expression (D) and food intake (E) were decreased in adult flies overexpressing *Dilp2* in IPCs (*Dilp2>Dilp2*) and overexpressing *insulin receptor* (*InR*) in *sNPFR1* neurons (*sNPFR1>InR^WT^*), while *sNPF* mRNA and food intake increased when *InR* was suppressed in *sNPFR1* neurons (*sNPFR1>InR^DN^*). (F) Fasting (at 24 h) reduces *Dilp3, and Dilp 5* mRNA but not *Dilp2* mRNA. Data are presented as means ± s.e.m. from three independent experiments. *P<0.05 (One-way ANOVA analysis).

The positive feedback regulation of sNPF signaling we have described to this point must occur alongside a system to negatively regulate sNPF signaling. Insulin, one of several anorexigenic hormones, inhibits food intake through AKT-mediated FoxO1 inactivation in the hypothalamus [27]. In *Drosophila*, neuronal overexpression of *Dilps* negatively regulates larval food intake [28]. To understand the inhibitory mechanism of insulin on food intake, we analyzed the phosphorylation of FOXO and the expression of *NPY* and *sNPF*. In the mouse hypothalamic GT1-7 cells, insulin treatment increased FoxO1 phosphorylation and decreased *NPY* mRNA while insulin combined with AKT inhibitor co-treatment slightly decreased FoxO1 phosphorylation and increased *NPY* expression (Figure 5A, 5B). Likewise, in fly neuronal BG2-c6 cells, insulin with AKT inhibitor co-treatment increased *sNPF* mRNA (Figure 5C). Thus, in both models AKT-mediated insulin signaling increased FOXO phosphorylation and suppressed *NPY* or *sNPF* expression.

We extended these results with analyses of *Drosophila* with insulin and insulin receptor transgenes. Compared to *Dilp2-Gal4* and *sNPFR1-Gal4* controls, *sNPF* mRNA and food intake were decreased when *Dilp2* was overexpressed in insulin producing cells (*Dilp2>Dilp2*) and when *insulin receptor* (*InR*) was overexpressed in *sNPFR1* expressing neurons (*sNPFR1>InR^WT^*) (Figure 5D, 5E). On the other hand, *sNPF* expression and food intake were increased when *InR* was suppressed by a dominant negative construct expressed in *sNPFR1* neurons (*sNPFR1>InR^DN^*) (Figure 5D, 5E). Fasting may contribute to *sNPF* expression and the propensity for food intake because fasting in the adult reduces the expression of several *Dilps* (Figure 5F), as previously observed to occur in *Drosophila* larvae [29].

Taken together, the results from mouse and *Drosophila* neuronal cells and from adult flies indicate that the insulin signaling negatively regulates *sNPF/NPY* expression and food intake.

### *Dyrk1a* TG Mice Regulate Food Intake through the FOXO-NPY Pathway

**Figure 6:**
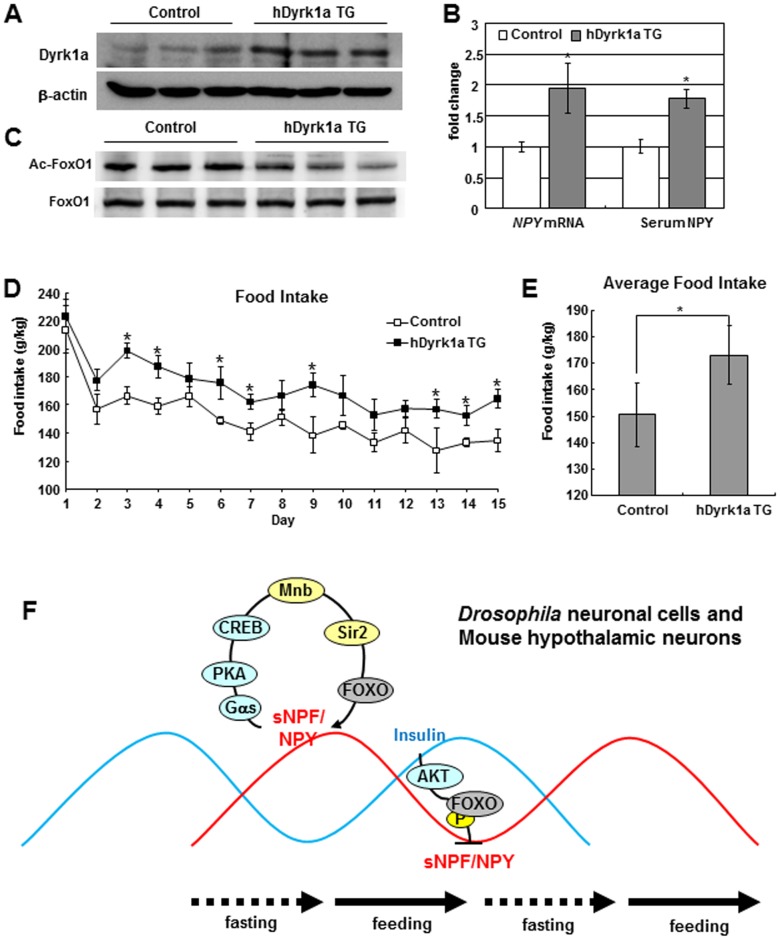
*hDyrk1a* transgenic mice regulate food intake through the FOXO-NPY pathway. (A, C) In the hypothalamus of *hDyrk1a* transgenic mice, Dyrk1a was increased and FoxO1 acetylation was reduced compared with those of the littermate control mice. (B) *NPY* mRNA from hypothalamus and serum NPY were increased in *hDyrk1a* transgenic mice. (D) Daily food intake was increased in *hDyrk1a* transgenic mice compared with the littermate controls. (E) Average food intake of *hDyrk1a* transgenic mice increased by 15%. Data are presented as means ± s.e.m. from three independent experiments. *P<0.05 (One-way ANOVA analysis). (F) The model of this study.

To evaluate these Mnb/Dyrk1a-Sir2-FOXO-NPY interactions and consequences in a mammalian animal model, we examined FoxO1 acetylation and NPY expression in the hypothalamus of transgenic mice containing the human *Dyrk1a* BAC clone (*hDyrk1a* TG). As expected, in the Western blot, Dyrk1a in the hypothalamus was increased in *hDyrk1a* TG mice compared to controls (Figure 6A). On the other hand, FoxO1 in the hypothalamus was less acetylated in *hDyrk1a* TG mice compared to controls (Figure 6C). Hypothalamic *NPY* mRNA as well as serum NPY levels were elevated in in *hDyrk1a* TG mice compared to controls (Figure 6B). Thus, mammalian Dyrk1a appears to regulate FoxO1 acetylation and NPY expression in the mouse hypothalamus, as we have observed for this system in *Drosophila* sNPFR1 neurons.

To assess whether mammalian *Dyrk1a* also regulates food intake as seen for the homolog *mnb* of *Drosophila*, we monitored food intake in seven-week-old *hDyrk1a* TG mice. Daily food consumption was increased in the transgenic mice compared to littermate controls (Figure 6D) and the average food intake of *hDyrk1a* transgenic mice was elevated by 15% (Figure 6E). Correspondingly, the *hDyrk1a* transgenic mice presented slightly increased mass (Figure S11). *Dyrk1a* thus appears to regulate food intake through the expression of NPY mediated by FOXO in a molecular pathway that is evolutionarily conserved in *Drosophila*.

## Discussion

The production of sNPF and NPY in sNPFnergic and hypothalamic neurons of flies and mammals respectively, is increased during fasting. These neuropeptides are secreted to produce paracrine and endocrine effects [24] but also feedback upon their synthesizing neurons where they respectively induce *mnb* and *Dyrk1a* gene expression through the PKA-CREB pathway (Figure 6F). This Mnb/Dyrk1a kinase phosphorylates and activates the Sir2/Sirt1 deacetylase, which in turn deacetylates and activates the FOXO transcription factor. Among its many potential targets, FOXO then increases *sNPF/NPY* mRNA expression. Negative controls modulate the positive feedback of sNPF/NPY. Feeding activates the insulin receptor-PI3K-AKT pathway. FOXO becomes phosphorylated and transcriptionally inactivated by translocation to the cytoplasm [30]. In this state the induction of *sNPF/NPY* by FOXO is decreased. Because sNPF and NPY are orexogenic, their positive feedback during fasting should reinforce the propensity for food intake whereas the negative regulation of *sNPF* and *NPY* mRNA during feeding condition would then contribute to satiety (Figure 6F).

FOXO family transcriptional factors are involved in metabolism, longevity, and cell proliferation [31]. FOXO is in part regulated in these processes by post-transcriptional modifications including phosphorylation and acetylation [30]. In many model systems, the ligand activated Insulin-PI3K-AKT pathway phosphorylates FOXO to inactivate this transcription factor by moving it to the cytoplasm. The cytoplasmic localization of FOXO is mediated by 14-3-3 chaperone proteins in *Drosophila* and mammals [32], [33]. FOXO may also be acetylated, as is FoxO1 of mice, by the CREB-binding protein (CBP)/p300 acetylase and this inhibits FOXO transcriptional function by suppressing its DNA-binding affinity. Such FoxO1 acetylation can be reversed by SirT1 to help activate the FoxO1 transcription factor [34]. Here we describe for *Drosophila* how dFOXO in sNPFR1 neurons regulates the expression of *sNPF* and food intake (Figure 4D, 4E). This mechanism parallels how hypothalamic FoxO1 regulates food intake through its control of orexigenic *NPY* and *Agrp* in rodents [12], [27]. Post-transcriptional modification of FOXO is central to these controls in both animals. *sNPF* and *NPY* expression is increased when FOXO is deacetylated by Sir2/Sirt1, while *sNPF* and *NPY* are decreased when FOXO is phosphorylated via the Insulin-PI3K-AKT pathway. Post-transcriptional modifications of FOXO proteins play a critical role for controlling food intake through the *sNPF* and *NPY* expression in flies and rodents.

Mnb/Dyrk1a has been described to participate in olfactory learning, circadian rhythm, and the development of the nervous system and brain [6]. Mnb and Dyrk1a proteins contain a nuclear targeting signal sequence, a protein kinase domain, a PEST domain, and a serine/threonine rich domain. The kinase domains are evolutionary well-conserved from flies to humans [35]. In Down syndrome (DS), chromosome 21 trisomy gives patients three copies of a critical region that includes the *Mnb/Dyrk1a*; trisomy of this region is associated with anomalies of both the nervous and endocrine systems [36]. DS patients often show high Body Mass Index due to the increased fat mass. Children with DS have elevated serum leptin coupled with leptin resistance, both of which contribute to the obesity risk common to DS patients [37], [38]. We now observe a novel function of Mnb/Dyrk1a that may underlay this metabolic condition of DS patients. Mnb/Dyrk1a regulates food intake in flies and mice. This is controlled by sNPF/NPY-PKA-CREB up-stream signaling and thus produces down-stream affects upon Sir2/Sirt1-FOXO-sNPF/NPY. Fasting not only increases the expression of *mnb*, but also of *sNPF*, suggesting that Mnb kinase activates a positive feedback loop where Sir2-dFOXO induces *sNPF* gene expression. Notably, fasting increases Sirt1 deacetylase activity and localizes FoxO1 to the nucleus in the orexogenic AgRP neurons of the mouse hypothalamus [15]. Increased dosage of *Dyrk1a* in DS patients may reinforce the positive feedback by NPY and disrupt the balance between hunger and satiety required to maintain a healthy body mass.

Insulin produced in the pancreas affects the hypothalamus to regulate feeding in mammals [1]. Insulin injected into the intracerebroventrical of the hypothalamus reduces food intake while inhibiting insulin receptors of the hypothalamic ARC nucleus causes hyperphasia and obesity in rodent models [39], [40]. Here we saw a similar pattern for *Drosophila* where overexpression of insulin-like peptide (*Dilp2*) at insulin producing neurons decreased food intake while food intake was increased by inhibiting the insulin receptor in sNPFR1 expressing neurons (Figure 5E). Likewise, during fasting, serum insulin and leptin levels are decreased in mammals [1], as is mRNA for insulin-like peptides of *Drosophila* [29], [41] (Figure 5F). Thus, the mechanism by which insulin and insulin receptor signaling suppresses food intake is conserved from fly to mammals in at least some important ways.

Previously, we reported how sNPF signaling regulates *Dilp* expression through ERK in IPCs and controls growth in *Drosophila* [3]. Here, we show that sNPF signaling regulates *mnb* expression through the PKA-CREB pathway in non-IPC neurons and controls food intake (Figure 1B, 1D–1G). Since sNPF works through the sNPFR1 receptor, sNPFR1 in IPCs and non-IPCs neurons might transduce different signals and thereby modulate different phenotypes. Four *Dilps* (*Dilp1*, *2*, *3*, and *5*) are expressed in the IPCs of the brain [42]. Interestingly, levels of *Dilp1* and *2* mRNA are reduced in the *sNPF* mutant, which has small body size [3], but here we find only *Dilp3* and *5* mRNA levels are reduced upon 24 h fasting. Likewise, only *Dilp5* is reduced when adult flies are maintained on yeast-limited diets [43]. In addition, *Dilp1* and *2* null mutants show slight reduced body weights but *Dilp3* and *Dilp5* null mutants do not [44]. These results suggest that *Dilp1* and *2* behave like a mammalian insulin growth factor for size regulation while *Dilp3* and *5* act like a mammalian insulin for the regulation of metabolism. However, in the long term starvation, *Dilp2* and *Dilp5* mRNA levels are reduced and *Dilp3* mRNA expression is increased [45].

During fasting, *sNPF* but not *sNPFR1* mRNA expression was increased in samples prepared from fly heads (Figure 4F and Figure S9C), which increases food intake. On the other hand, in feeding, the high level of insulin signaling reduced *sNPF* but not *sNPFR1* mRNA expression and suppressed food intake (Figure 5D and 5E, Figure S9D). Interestingly, in the antenna of starved flies, *sNPFR1* but not *sNPF* mRNA expression is increased and induces presynaptic facilitation, which resulted in effective odor-driven food search. However, high insulin signaling suppresses *sNPFR1* mRNA expression and prevents presynaptic facilitation in DM1 glomerulus [46]. These results indicate that starvation-mediated or insulin signaling-mediated sNPF-sNPFR1 signaling plays a critical role in *Drosophila* feeding behavior including food intake and food search even though the fine tuning is different.

In this study, we present a molecular mechanism for how sNPF and NPY regulate food intake in *Drosophila* and mice. We describe a system of positive feedback regulation for sNPF and NPY signaling that increases food intake and a mode of negative regulation for sNPF and NPY by the insulin signaling that suppresses food intake. Modifications of the FOXO protein play a critical role for regulating *sNPF* and *NPY* expression, resulting in the control of food intake.

## Materials and Methods

### *Drosophila* Culture and Stocks

*Drosophila melanogaster* were cultured and at 25°C on standard cornmeal, yeast, sugar, agar diet. Wild-type *Canton-S*, *w^-^*, and *UAS-CREB-DN* were obtained from the Bloomington stock center. *sNPF^c00448^* was obtained from the Harvard stock center (Exelixis stock collection). *UAS-sNPF, UAS-2XsNPF, UAS-sNPF-Ri, UAS-sNPFR1, UAS-sNPFR1-DN and sNPF-Gal4* transgenic flies were described in our previous reports [2], [3], [16]. The *sNPFR1-Gal4* construct was generated from a 2.5 kb genomic DNA fragment of the 5′-untranslated region of the *sNPFR1* gene. The full-length coding sequence of *Drosophila minibrain-H* (*mnb*, *CG 42273*) was subcloned into the *pUAS* vector to generate the *pUAS-mnb* construct. *sNPFR1-Gal4* and *UAS-mnb* transgenic flies were obtained by the P-element-mediated germ line transformation [47]. *mnb^G1767^*, an EP line for *minibrain*, was purchased from the GenExel, Inc. (KAIST, Korea). *UAS-sNPFR1-Ri* (VDRC9379), *UAS-mnb-Ri* (VDRC28628), *UAS-Sir2-Ri* (VDRC23201) and *UAS-FOXO-Ri* (VDRC106097) were obtained from the Vienna *Drosophila* RNAi Center (VDRC). *Dilp2-Gal4*, *UAS-Gαs-Ri*, *UAS-PKA-DN* (a dominant-negative form of PKA), *UAS-Sir2* transgenic flies were described previously [42], [48], [49], [50], [51]. To express these UAS lines, UAS-Gal4 system was used [52]. For minimizing the genetic background effect among tested *Drosophila* lines, all stocks were crossed with *w-* and then crossed to the second (*w-; Bc, Elp/CyO*) or third (*w-; D/TM3, Sb*) chromosome balancers, respectively. For making double mutants, *w-; T(2:3) Ap^Xa^/CyO; TM3* was crossed with the flies containing *UAS-X* transgene to produce *w-; UAS-X/CyO; +/TM3*. Then, *w-; +/CyO; UAS-Y/TM3* flies generated by the similar way were crossed with *w-; UAS-X/CyO; +/TM3* to produce *w-; UAS-X/CyO; UAS-Y/TM3*.

### Cell Culture, Stimulation, and Transfection

*Drosophila* BG2-c6 cells established by the single colony isolation of primary cells derived from the third instar larval central nervous system. This cell line synthesizes acetylcholine and expresses insect neuron specific glycans and a RNA-binding protein Elav [19]. BG2-c6 cells purchased from the *Drosophila* Genomics Resource Center (DGRC, Indiana University) were maintained at 26°C in Schneider medium supplemented with 10% bovine calf serum. Immortalized GT1-7 mouse hypothalamic neurons [22] were cultured in 4.5 g/l glucose Dulbecco's modified Eagle's medium (DMEM) supplemented with 10% fetal bovine serum, 2% of l-glutamine, 100 µU/ml penicillin and 100 µg/ml streptomycin in 5% CO_2_ at 37°C. The culture medium was changed every 2–3 days. Before peptide treatments, cells were starved for 8 h in the serum-free medium containing 0.5% BSA and pretreated with a chemical inhibitor or vehicle. PKA inhibitor H89 (10 µM, Calbiochem), ERK-specific kinase MEK inhibitor PD98059 (10 µM, Calbiochem), PKC inhibitor Chelerythrine chloride (1 µM, Sigma) were used. NPY1R inhibitor BIBO3304 (10 nM), NPY2R inhibitor BIIE0246 (50 nM), NPY5R inhibitor CGP71683 (1 µM) and Sirt1 inhibitor EX527 (10 µM) were purchased from Tocris. Then, cells were treated with 100 nM synthetic 19 amino acids sNPF2 or 100 nM human NPY 1–36 peptide (Sigma). For transfection, cells were cultured in the growth medium without antibiotics and transfected with small interfering RNA (siRNA) using Lipofectamine 2000 reagent (Invitrogen). *Gαs* and *Gαi* siRNA constructs were designed by the BLOCK-iT RNAi Designer and *Dyrk1a* siRNA was purchased from Invitrogen. The sequences of siRNA are caggauauucuucggugccguguuu for Gαs and cggcgggauacuaucuaaauucgcu for *Gαi*. The BLOCK-iT Fluorescent Oligo, which is a fluorescent-labeled dsRNA oligomer, was used as the non-targeting siRNA control. For the overexpression mouse *Dyrk1a*, a full-length *mDyrk1a* cDNA was cloned to pCDNA3.1 (Invitrogen).

### *Drosophila* Food Intake Assay

We measured food intake of *Drosophila* in two ways. The CAFE assay [17] was performed with 3 day-old adult male flies. Twelve hours before the assay, ten flies were placed in the CAFE device [17] containing 5% sucrose solution in calibrated glass micropipettes (VWR, West Chester, PA). At time zero, the micropipettes with 5% sucrose solution were replaced and the amount of liquid consumed was measured every 6 h. A colorimetric food intake assay was modified from published methods [2], [53]. Since flies had most fed color food in the crop during first 30 min and started to excrete from 1 h (Figure S4C, S4D) [54], flies were starved in PBS-containing vials for 2 h and fed for 30 min in vials containing 0.05% Bromophenol Blue dye and 10% sucrose in yeast paste. Then, the flies were frozen, homogenized in PBS, and centrifuged twice for 25 min each. The supernatant was measured at 625 nm. Each experiment consisted of 20 flies, and the assay was repeated at least three times.

### Mouse Food Intake Assay

*Dyrk1a* transgenic mice expressed the human *Dyrk1a* BAC clone in the *C57BL/6* background [55]. Seven weeks-old male *Dyrk1a* TG and littermate control mice were used in the experiments (n = 7). The mice were housed individually in the standard plastic rodent cages. They were maintained at 22±2°C in a room with a 12-hour light/dark cycle and habituated to frequent handling. Food intake and body weight were measured within 30 min before the light turned on and off. Drinking water was available at all times. Food intake data were corrected with body weight.

Animal care and all experiments were conducted according to KRIBB Guidelines for the Care and Use of Laboratory Animals and Inje University Council.

### *Drosophila* Starvation

Twenty *w-* female flies were starved overnight and fed for 2 h for the physiological synchronization. Then, starvations for the experiment were started. The heads from starved flies were collected for the Quantitative RT-PCR analysis. The experiments were repeated three times.

### Measurement of *Drosophila* Body Weight

Eggs laid by five female flies for 6 h at 25°C were cultured to avoid over-crowding and lack of nutrition. For weight of individual fly, over 50 three day-old adult male flies were measured with the balancer (METTLER AJ100) and divided with the number of flies. At least three experiments were performed in each assay.

### Quantitative RT–PCR Analysis

Adult heads from 20 flies were collected for RNA preparation. Total RNA was extracted using the easy-BLUE (TM) reagent (iNtRON biotechnology). All RNA samples were treated with RNase-free DNase (Promega). cDNA was synthesized using a SuperScript III First-Strand Synthesis System (Invitrogen). For quantitative RT-PCR analysis, ABI Prism 7900 Sequence Detection System (Applied Biosystems) and SyberGreen PCR Core reagents (Applied Biosystems) were used. mRNA levels were expressed as the relative fold change against the normalized *rp49* mRNA. The comparative cycle threshold (Ct) method (User Bulletin 2, Applied Biosystems) was used to analyze the data. All experiments were repeated at least three times. The statistical significance was tested by Microsoft Excel-based application for the student *t*-test statistical analysis. Primers used in the RT-PCR analyses were listed in Table S2.

### Generation of the Minibrain and sNPFR1 Antisera and Immunostaining in the Adult Brain of *Drosophila*

Minibrain antiserum was generated by the immunization of rabbits with the synthetic peptide (CQHRVRNWPTNGNQ) corresponding to the N-terminal sequence (75–88) of the Minibrain-H protein. Antiserum against sNPFR1 was generated by the immunization of rat with the synthetic peptide (GEAIGAGGGAELGRRIN) corresponding to the C-terminal sequence (585–600) of the sNPFR1 protein. For immunostaining, adult brain from newly eclosed flies (3 day old) was dissected in PBS, fixed in 4% paraformaldehyde, and blocked in 5% BSA and 5% normal goat serum. Primary antibodies were incubated two days at 4°C and secondary antibodies were incubated for 2 h at room temperature. The tissues were mounted in the DABCO solution (70% glycerol, 2.5% DABCO, Sigma, St Louis, MO) and fluorescence images were acquired by FluoView confocal microscope (Olympus). sNPF (1∶200), sNPFR1 (1∶200), and Minibrain (1∶200) primary antibodies, and anti-rat IgG Alexa 488, anti-rabbit IgG Alexa 488, or Alexa 594 (1∶200, Molecular Probes) and anti-guinea pig Cy5 (1∶200, Jackson ImmunoResearch) secondary antibodies were used.

### Western Blot Analysis

The cells were lysed by the Lysis buffer (Cell signaling) containing NaF, PMSF and Na_3_VO_4_. Total cell lysates were immunoprecipitated with Sirt1 antibody (Cell signaling) and protein A-agarose (Pierce). The immunoprecipitates were washed three times with Lysis buffer and solubilized in the SDS sample buffer (63.5 mM Tris-HCl; pH 6.8, 2% w/v SDS, 10% glycerol, 50 mM DTT, 0.01% w/v bromphenol blue). Western blot analyses were performed as described previously [2]. Phospho-CREB, phospho-Threonine, FoxO1 (1∶1000, Cell signaling), Ac-FKHR (1∶1000, Santa Cruze), β-actin (1∶3000, Abcam) primary antibodies, and horseradish peroxidase-conjugated anti-rabbit IgG (1∶5000, Santa Cruze) and anti-mouse IgG secondary antibody (1∶5000, Sigma) were used.

### cAMP Assay

Intracellular cAMP was measured with the cAMP Biotrak Enzyme Immunoassay Kit (GE Healthcare) by the manufacturer's instruction. Briefly, samples were incubated with anti-cAMP antibody, which was immobilized in the secondary antibody coated micro-plates. Following enzyme substrate conversion, an optical density was measured at 450 nm with microplate reader (Fluostar Optima, BMG labtech). cAMP concentration was expressed as the cAMP pM per mg of protein and converted to the fold change relative to the basal control value.

### ChIP-on-chip and ChIP–PCR Analysis

About 250 of 3-day-old W[DAH] female flies were collected after 12 h starvation. Then, flies were homogenized and cross-linked in 1X PBS containing 1% formaldehyde. The ChIP protocol was performed as described in Teleman et al. [56]. Immunoprecipitation was performed using Dynal protean G beads (Invitrogen) and anti-dFOXO antibody (a gift from Heather Broihier). Purified DNA was amplified and labeled following Affymetrix ChIP Assay Protocol. Drosophila Tiling 2.0R Array was used to detect dFOXO binding enrichment. For ChIP-PCR analysis, about 10^8^ of BG2-c6 cells were treated with sNPF2 peptide as described above. sNPF-treated and untreated cells were cross-linked with 1% formaldehyde. After immunoprecipitation with the CREB antibody (Cell signaling) and Protein A Sepharose CL-4B (GE Healthcare), quantitative RT-PCR analysis was performed using input DNA and immunoprecipitated DNA for the CREB binding site in the *mnb* promoter region and the 3^rd^ axon of *Actin5C*.

### Statistics

Values in the paper are presented as means ± s.e.m. Statistical significant of all data were evaluated by the One-way ANOVA test (GraphPad Prism software). *P*<0.05 was accepted as statistically significant.

## Supporting Information

Figure S1 (A) *mnb* mRNA expression levels of Figure 1A and *UAS* controls. (B) Relative food consumption of Figure 1B and *UAS* controls. (C) Relative food consumption of Figure 3C, Figure 4E, and *UAS* controls.
(TIF)

Figure S2 *sNPFR1-Gal4* expression was detected by *sNPFR1-Gal4>UAS-DsRed* (*sNPFR1>DsRed*) in the fly adult brain. (A-D) In the anterior focal planes, *sNPFR1>DsRed* was detected in the optic lobes (OL, A), insulin producing cells (IPCs, C), mushroom body (MB, B), and subesophageal ganglions (SOG, D). (E, F) In the posterior focal planes, *sNPFR1>DsRed* was detected in the median neurons above esophagus (E, dot box; F, asterisk). Scale bars are 100 µm (A, E), 50 µm (B, D, F) and 30 µm (C).
(TIF)

Figure S3 (A) Western blots with the Mnb antibody in the *sNPF-Gal4* control, *sNPF* overexpression (*sNPF>2xsNPF*), *sNPF^c00448^* mutant, *sNPFR1-Gal4* control, *sNPFR1* overexpression (*sNPFR1>sNPFR1*), and *sNPFR1* inhibition (*sNPFR1>sNPFR1-Ri*). (B-F) Numbers of strong Mnb expression neurons (asterisks) are similar in the *sNPFR1-Gal4* control, *sNPFR1* overexpression (*sNPFR1>sNPFR1*), *sNPFR1* inhibition (*sNPFR1>sNPFR1-Ri*), and *sNPF^c00448^* mutant. Scale bars are 100 µm.
(TIF)

Figure S4 (A) The *mnb* genomic organization. Open boxes represent exons, the triangle shows the p-element insertion site in *mnb^G1767^*, and an arrow indicates the transcriptional initiation of the *mnb H* isoform containing the longest coding sequences among *mnb* isoforms. *mnb* deletion mutants (*mnb^d305^* and *mnb^d419^*) were generated by imprecise excisions of the inserted p-element. (B) *mnb* mRNA expression levels in the *mnb* overexpression (*sNPFR1>mnb*), inhibition (*sNPFR1>mnb-Ri*), and *mnb^G1767^* mutant. (C) Western blot with the Mnb antibody in the *w-* control and *mnb^G1767^* mutant. (D) *mnb* overexpression (*sNPFR1>mnb*) increased the body weight compared with the *sNPFR1-Gal4* control whereas *mnb* suppression (*sNPFR1>mnb-Ri, mnb^G1767^*) decreased the body weight. (E) Amount of food intake by the normalized to body mass and to the number of flies. Data are presented as means ± s.e.m. from three independent experiments. *P<0.05 (One-way ANOVA analysis).
(TIF)

Figure S5 Adult specific food intake assay using the *tubGal80ts* inducible system. (A) In the 22°C permissive temperature condition in which *tubGal80ts* suppress *sNPFR1-Gal4* expression, *mnb* overexpression (*sNPFR1-Gal4+tubGal80ts>mnb, sNPFR1-Gal4+tubGal80ts>2Xmnb*) and *mnb* inhibition (*sNPFR1-Gal4+tubGal80ts>mnb-Ri*) flies did not change the amount of food intake compared with the control flies (*sNPFR1-Gal4;tub-Gal80ts*). (B) In the 30°C restrictive temperature in which *tubGal80ts* cannot suppress *sNPFR1-Gal4*, the *mnb* overexpression increased food intake compared with the control and the *mnb* inhibition suppressed food intake. Data are presented as means ± s.e.m. from three independent experiments. *P<0.05 (One-way ANOVA analysis).
(TIF)

Figure S6 (A) Expression levels of *sNPF* and *sNPFR1* in *Drosophila* BG2-C6 cells after sNPF treatment. (B) cAMP level in *Drosophila* BG2-c6 cells after sNPF treatment. (C) cAMP level in mouse GT1-7 cells after NPY treatment.
(TIF)

Figure S7 Levels of *mnb* mRNA expression (A), relative food consumption (B), and *sNPF* mRNA expression (C) in *Gal4* and *UAS* controls used in this study.
(TIF)

Figure S8 Promoter analysis of *mnb* genes from twelve *Drosophila* species reveals that the cAMP-response element (CRE), which is TGACGTCA, was conserved in *Drosophila* species including *D. melanogaster* (Adapted and modified from UCSC Genome Browser at http://genome.ucsc.edu).
(TIF)

Figure S9 (A, B) RT-PCR analysis of *sNPFR1*, *mnb*, *Sir2*, and *dFOXO* mRNA in the *sNPFR1-Gal4*, *sNPFR1>sNPFR1*, *sNPFR1>mnb*, *sNPFR1>Sir2*, and *sNPF1>dFOXO* overexpression and in the *sNPFR1>sNPFR1-Ri*, *sNPFR1>mnb-Ri*, *sNPFR1>Sir2-Ri*, and *sNPFR1>dFOXO-Ri* inhibition. (C) *sNPFR1* expression during fasting. (D) *sNPFR1* mRNA expression was not changed in *Dilp2>Dilp2* compared to the *Dilp2-Gal4* control and in *sNPFR1>InR* and *sNPFR1>InR^DN^* compared to the *sNPFR1-Gal*4 control.
(TIF)

Figure S10 Promoter analysis of *sNPF* genes from twelve *Drosophila* species reveals that the dFOXO binding site, which is RWWAACA, was conserved in five *Drosophila* species including *D. melanogaster* (Adapted and modified from UCSC Genome Browser at http://genome.ucsc.edu).
(TIF)

Figure S11 *hDyrk1a* transgenic mice showed slightly increased body weight. Data are presented as means ± s.e.m. *P<0.05.
(TIF)

Table S1 *mnb* expression in the DNA microarray analysis.
(DOC)

Table S2 PCR primer sequences in this study.
(DOC)
