# Supplementary figures and images for: Minibrain/Dyrk1a Regulates Food Intake through the Sir2-FOXO-sNPF/NPY Pathway in Drosophila and Mammals
Source: PLoS Genet. 2012 Aug 2;8(8):e1002857. doi: 10.1371/journal.pgen.1002857 (PMC3410862; doi:10.1371/journal.pgen.1002857)

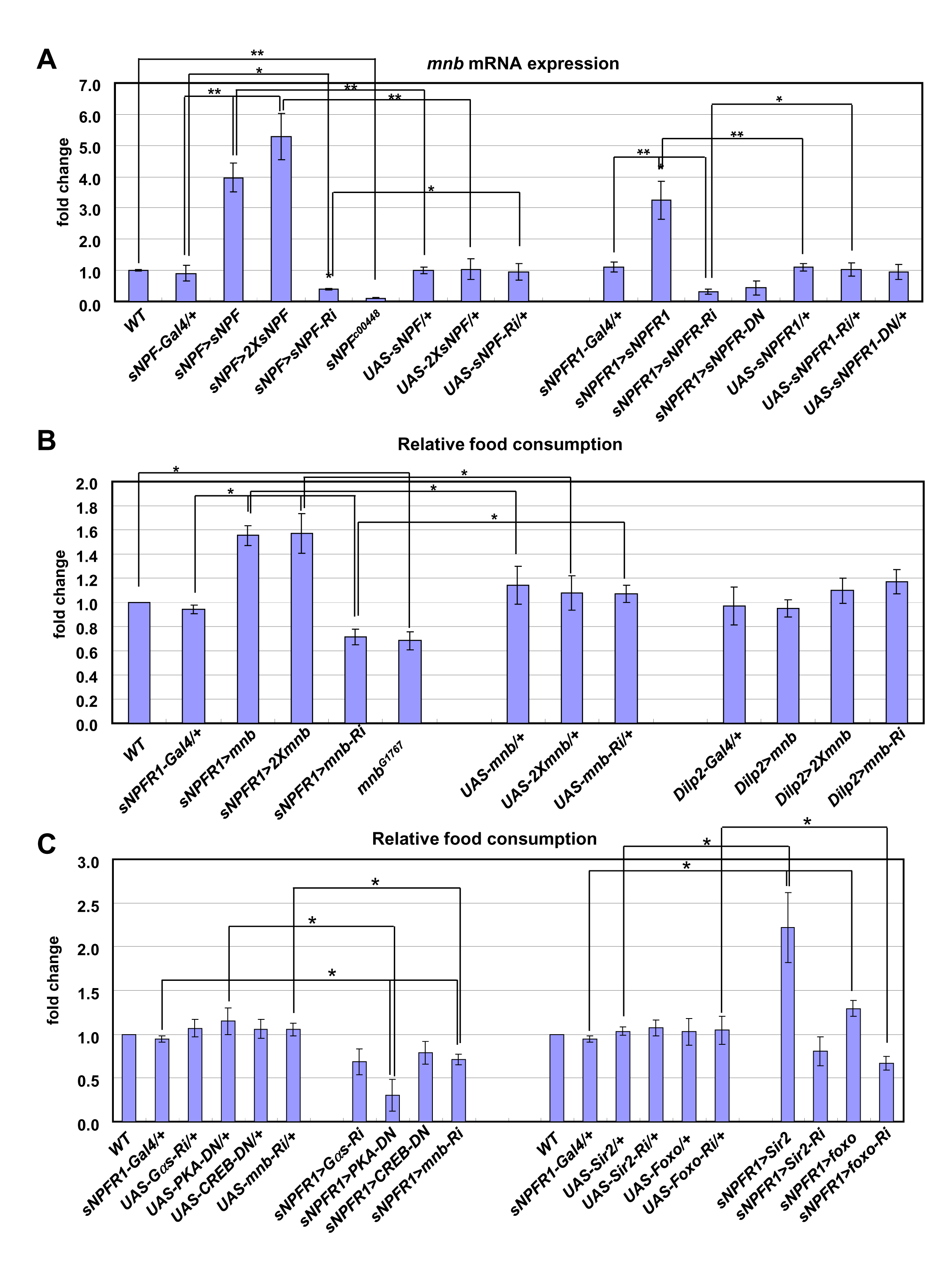

Supplement: Figure S1 — (A) mnb mRNA expression levels of Figure 1A and UAS controls. (B) Relative food consumption of Figure 1B and UAS controls. (C) Relative food consumption of Figure 3C, Figure 4E, and UAS controls. (TIF) [file pgen.1002857.s001.tif]

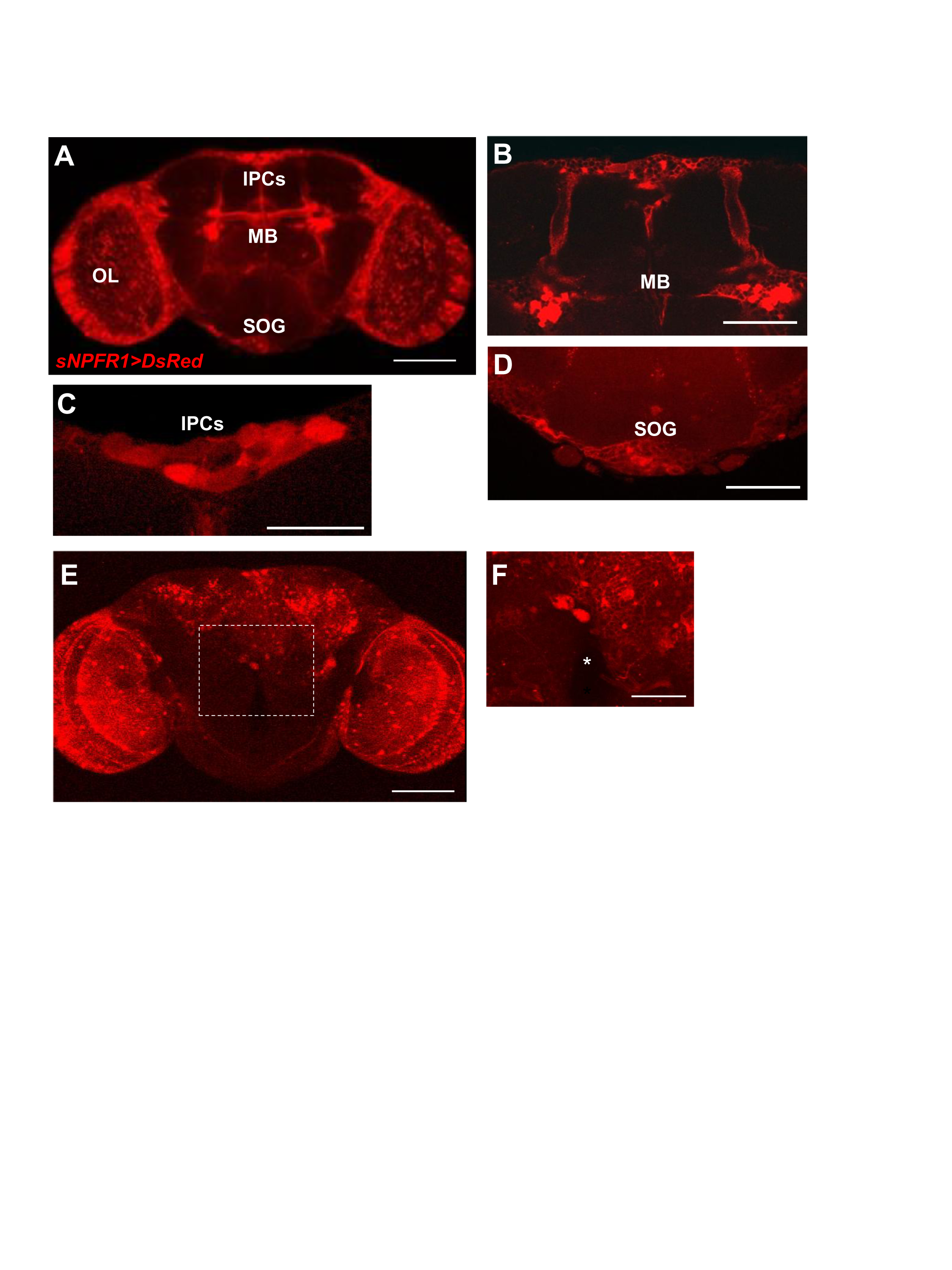

Supplement: Figure S2 — sNPFR1-Gal4 expression was detected by sNPFR1-Gal4>UAS-DsRed (sNPFR1>DsRed) in the fly adult brain. (A-D) In the anterior focal planes, sNPFR1>DsRed was detected in the optic lobes (OL, A), insulin producing cells (IPCs, C), mushroom body (MB, B), and subesophageal ganglions (SOG, D). (E, F) In the posterior focal planes, sNPFR1>DsRed was detected in the median neurons above esophagus (E, dot box; F, asterisk). Scale bars are 100 µm (A, E), 50 µm (B, D, F) and 30 µm (C). (TIF) [file pgen.1002857.s002.tif]

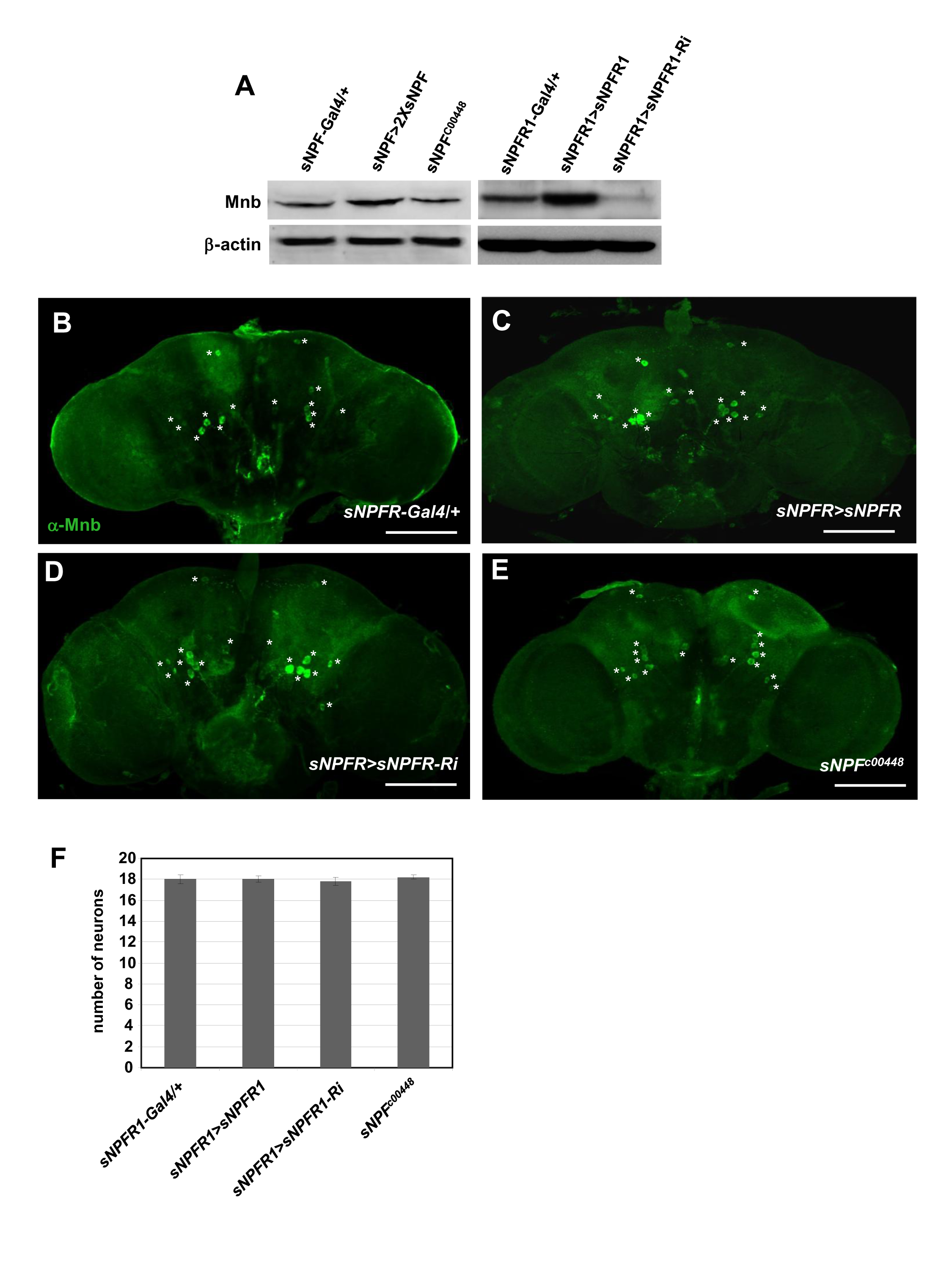

Supplement: Figure S3 — (A) Western blots with the Mnb antibody in the sNPF-Gal4 control, sNPF overexpression (sNPF>2xsNPF), sNPFc00448 mutant, sNPFR1-Gal4 control, sNPFR1 overexpression (sNPFR1>sNPFR1), and sNPFR1 inhibition (sNPFR1>sNPFR1-Ri). (B-F) Numbers of strong Mnb expression neurons (asterisks) are similar in the sNPFR1-Gal4 control, sNPFR1 overexpression (sNPFR1>sNPFR1), sNPFR1 inhibition (sNPFR1>sNPFR1-Ri), and sNPFc00448 mutant. Scale bars are 100 µm. (TIF) [file pgen.1002857.s003.tif]

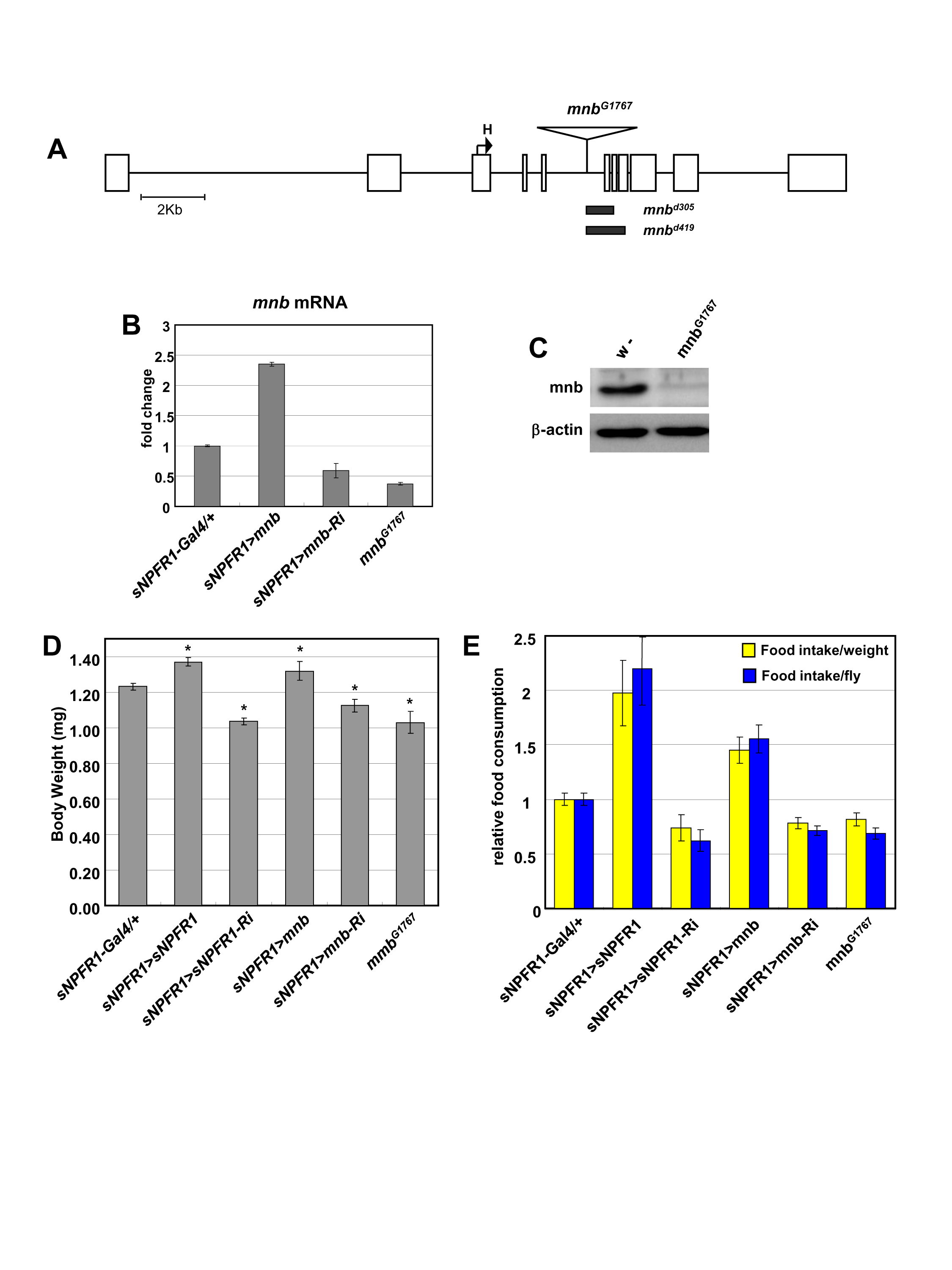

Supplement: Figure S4 — (A) The mnb genomic organization. Open boxes represent exons, the triangle shows the p-element insertion site in mnbG1767, and an arrow indicates the transcriptional initiation of the mnb H isoform containing the longest coding sequences among mnb isoforms. mnb deletion mutants (mnbd305 and mnbd419) were generated by imprecise excisions of the inserted p-element. (B) mnb mRNA expression levels in the mnb overexpression (sNPFR1>mnb), inhibition (sNPFR1>mnb-Ri), and mnbG1767 mutant. (C) Western blot with the Mnb antibody in the w- control and mnbG1767 mutant. (D) mnb overexpression (sNPFR1>mnb) increased the body weight compared with the sNPFR1-Gal4 control whereas mnb suppression (sNPFR1>mnb-Ri, mnbG1767) decreased the body weight. (E) Amount of food intake by the normalized to body mass and to the number of flies. Data are presented as means ± s.e.m. from three independent experiments. *P<0.05 (One-way ANOVA analysis). (TIF) [file pgen.1002857.s004.tif]

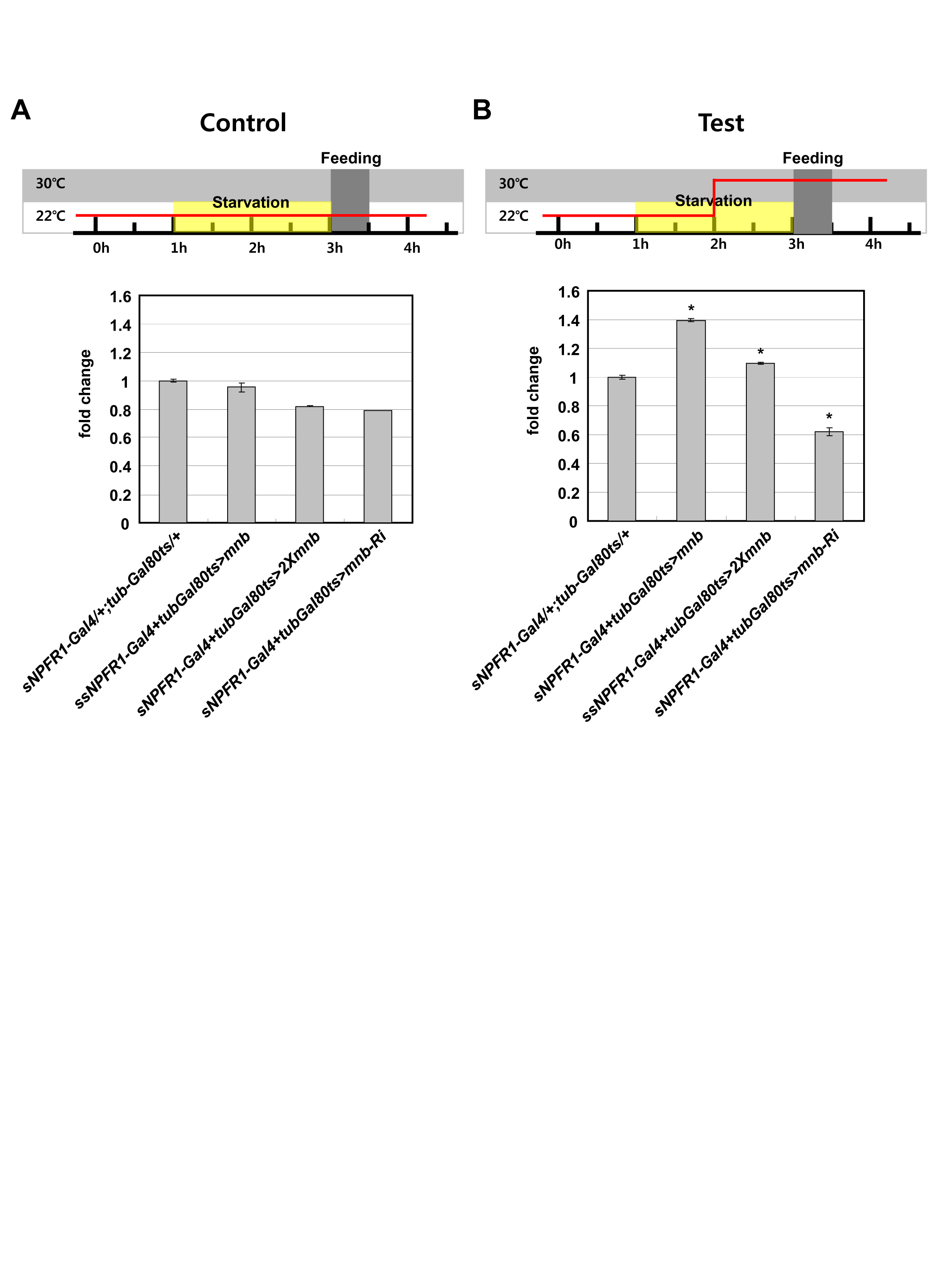

Supplement: Figure S5 — Adult specific food intake assay using the tubGal80ts inducible system. (A) In the 22°C permissive temperature condition in which tubGal80ts suppress sNPFR1-Gal4 expression, mnb overexpression (sNPFR1-Gal4+tubGal80ts>mnb, sNPFR1-Gal4+tubGal80ts>2Xmnb) and mnb inhibition (sNPFR1-Gal4+tubGal80ts>mnb-Ri) flies did not change the amount of food intake compared with the control flies (sNPFR1-Gal4;tub-Gal80ts). (B) In the 30°C restrictive temperature in which tubGal80ts cannot suppress sNPFR1-Gal4, the mnb overexpression increased food intake compared with the control and the mnb inhibition suppressed food intake. Data are presented as means ± s.e.m. from three independent experiments. *P<0.05 (One-way ANOVA analysis). (TIF) [file pgen.1002857.s005.tif]

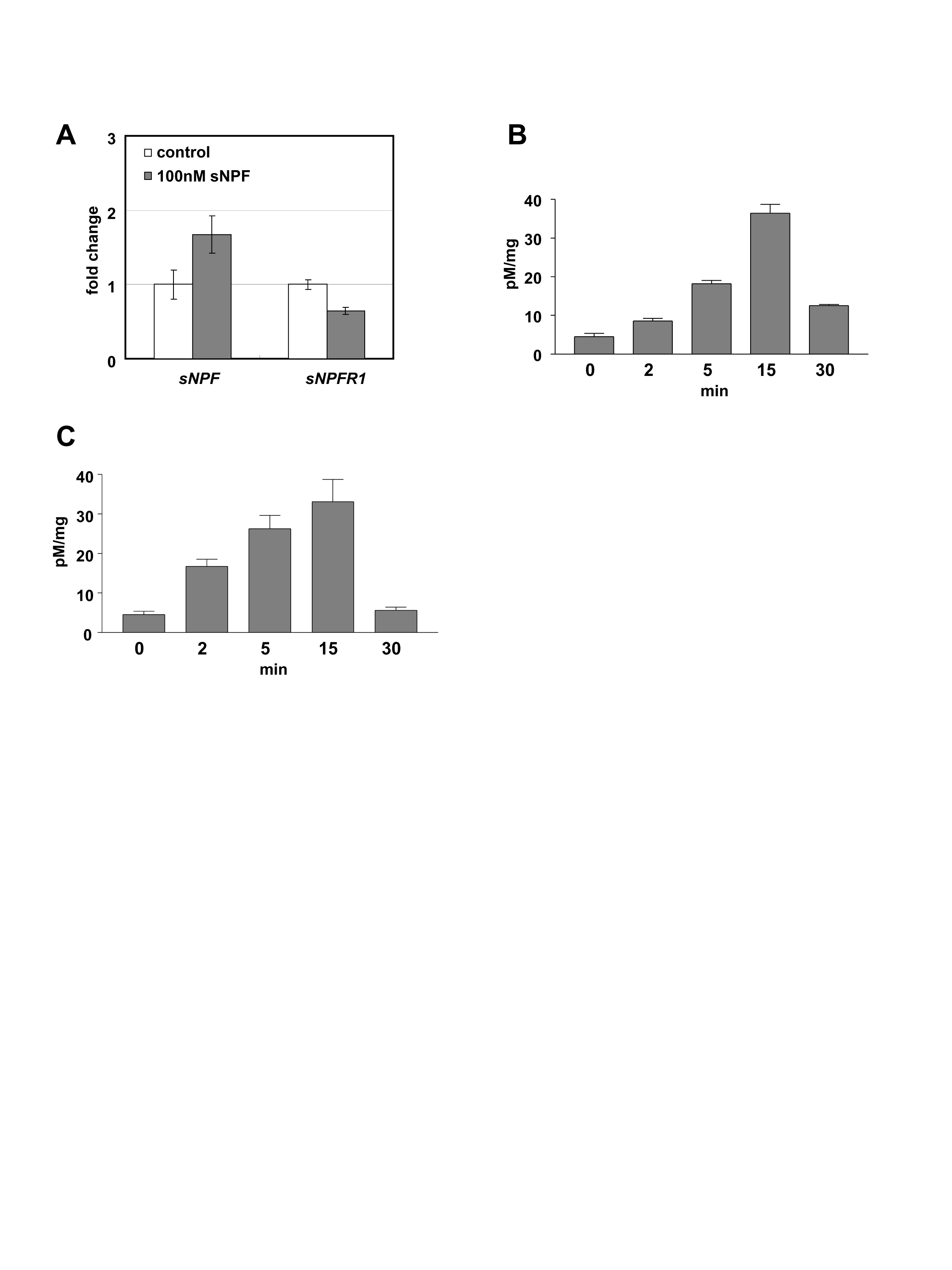

Supplement: Figure S6 — (A) Expression levels of sNPF and sNPFR1 in Drosophila BG2-C6 cells after sNPF treatment. (B) cAMP level in Drosophila BG2-c6 cells after sNPF treatment. (C) cAMP level in mouse GT1-7 cells after NPY treatment. (TIF) [file pgen.1002857.s006.tif]

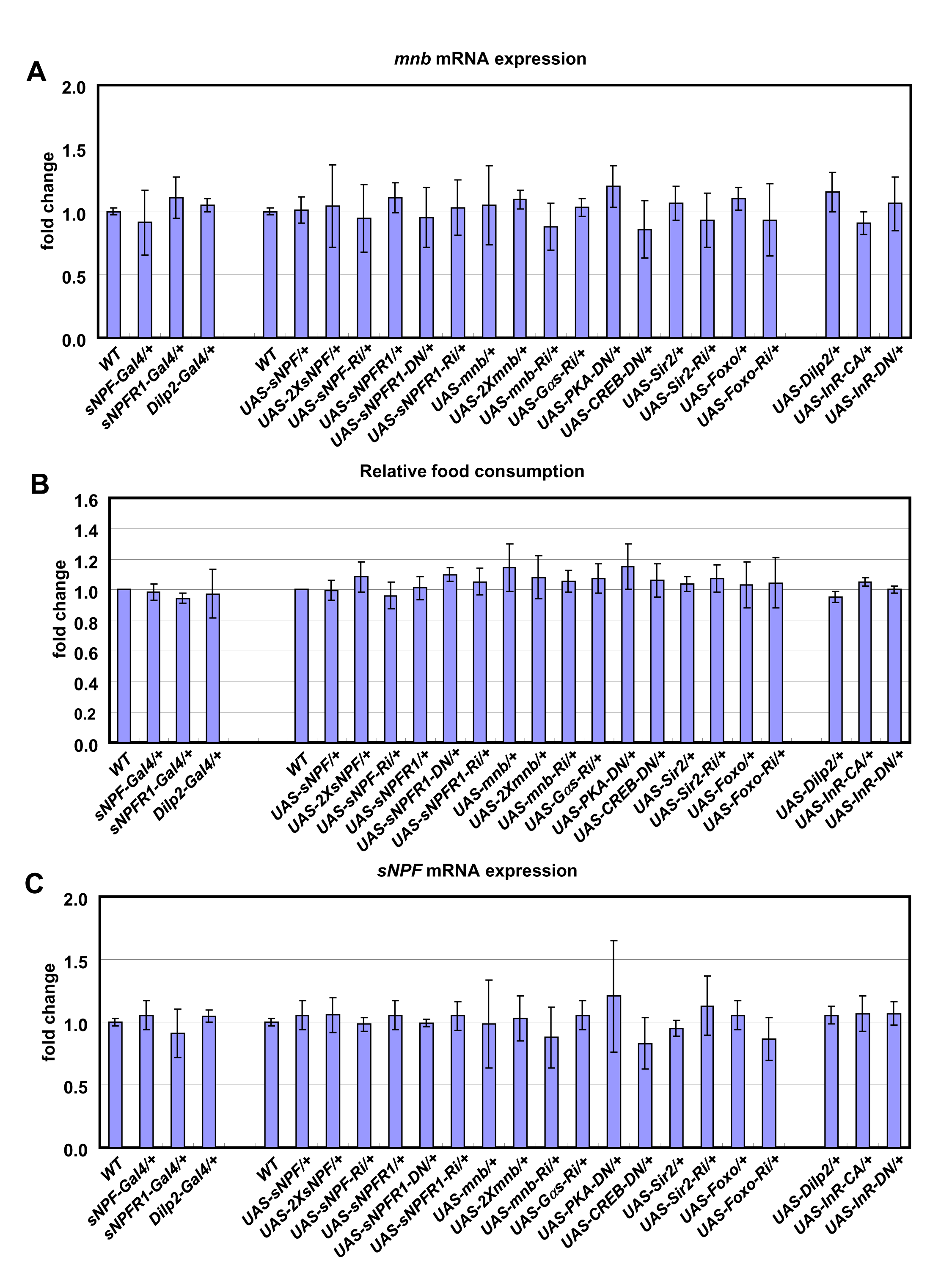

Supplement: Figure S7 — Levels of mnb mRNA expression (A), relative food consumption (B), and sNPF mRNA expression (C) in Gal4 and UAS controls used in this study. (TIF) [file pgen.1002857.s007.tif]

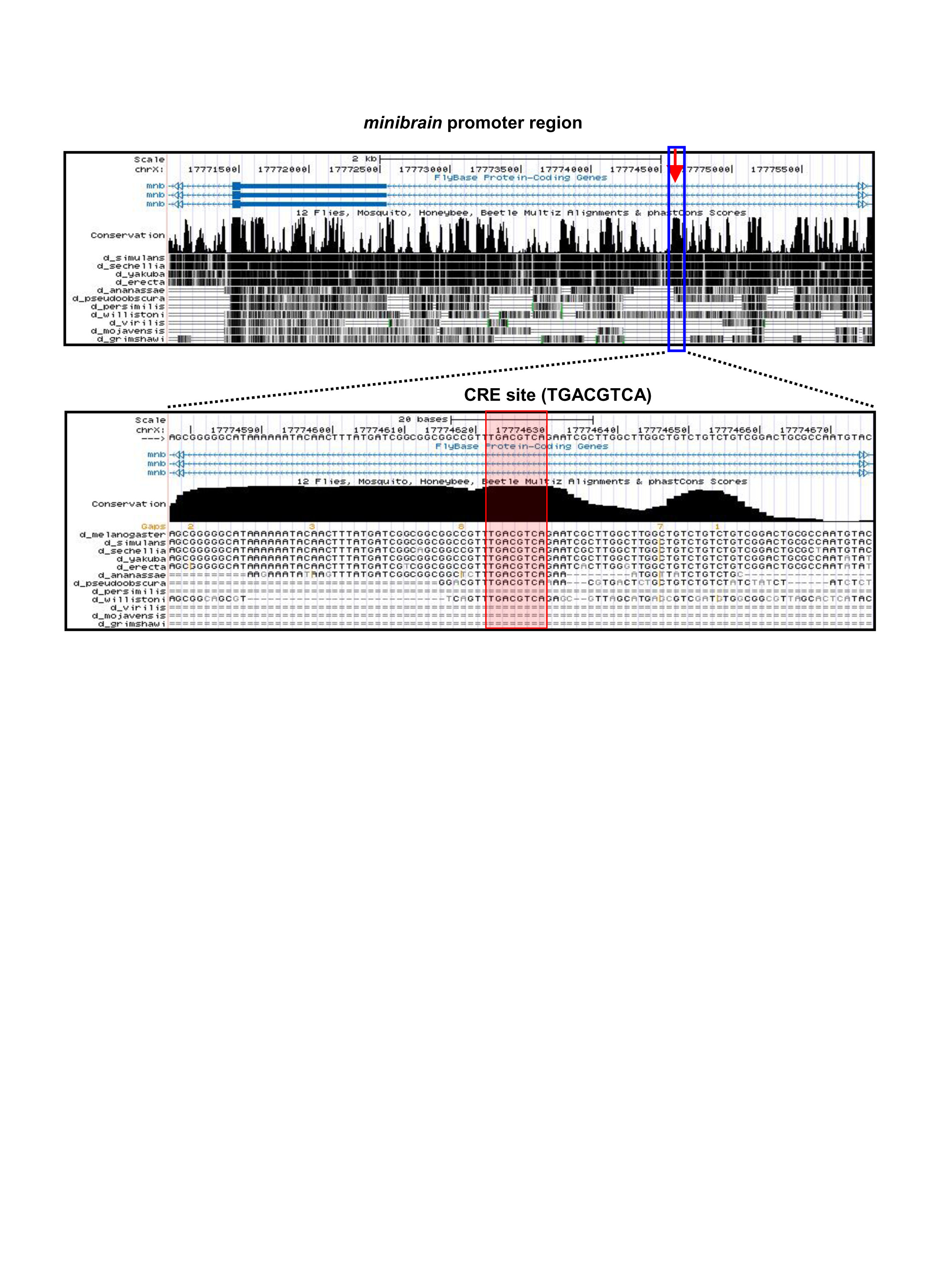

Supplement: Figure S8 — Promoter analysis of mnb genes from twelve Drosophila species reveals that the cAMP-response element (CRE), which is TGACGTCA, was conserved in Drosophila species including D. melanogaster (Adapted and modified from UCSC Genome Browser at http://genome.ucsc.edu). (TIF) [file pgen.1002857.s008.tif]

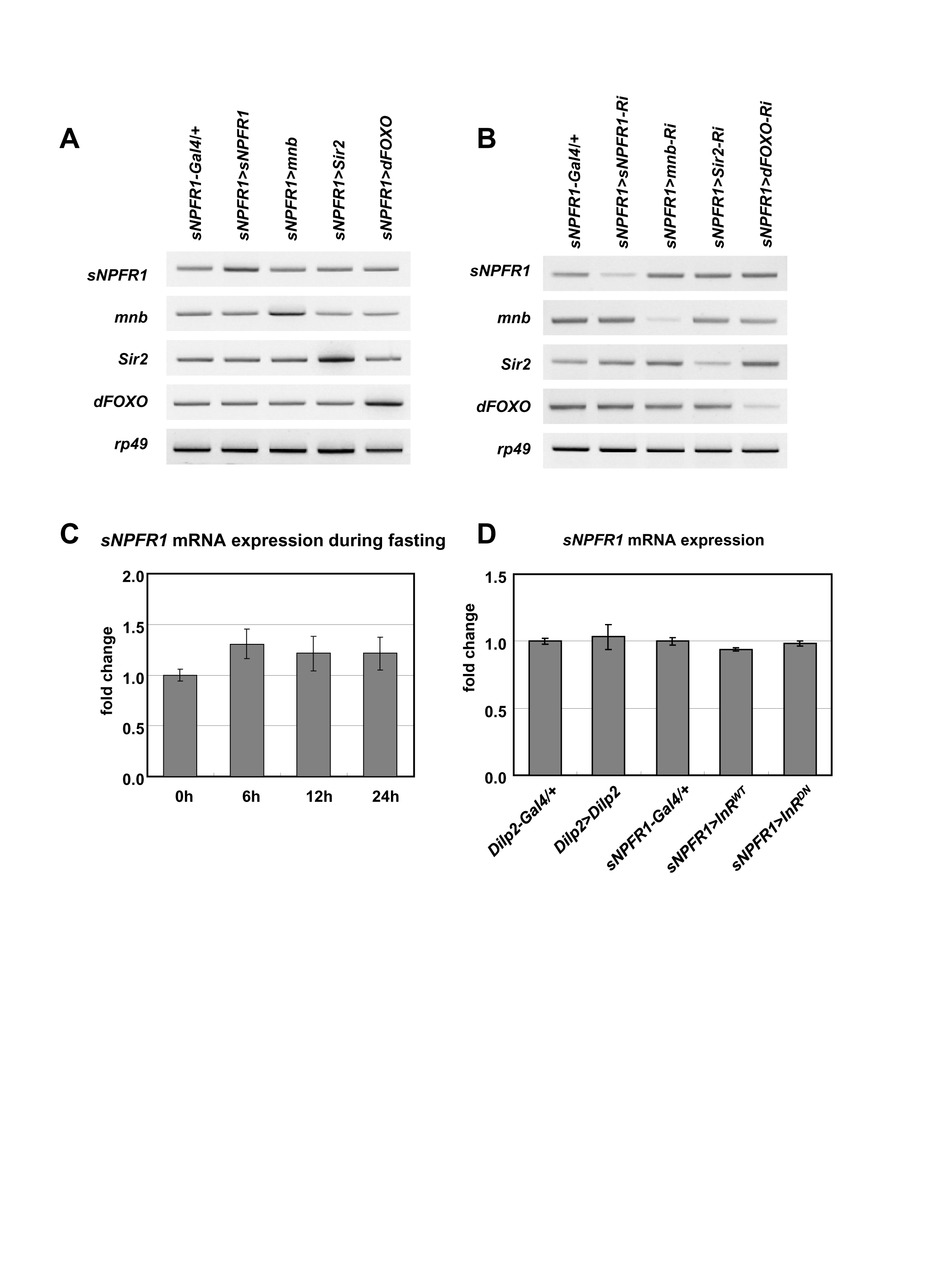

Supplement: Figure S9 — (A, B) RT-PCR analysis of sNPFR1, mnb, Sir2, and dFOXO mRNA in the sNPFR1-Gal4, sNPFR1>sNPFR1, sNPFR1>mnb, sNPFR1>Sir2, and sNPF1>dFOXO overexpression and in the sNPFR1>sNPFR1-Ri, sNPFR1>mnb-Ri, sNPFR1>Sir2-Ri, and sNPFR1>dFOXO-Ri inhibition. (C) sNPFR1 expression during fasting. (D) sNPFR1 mRNA expression was not changed in Dilp2>Dilp2 compared to the Dilp2-Gal4 control and in sNPFR1>InR and sNPFR1>InRDN compared to the sNPFR1-Gal4 control. (TIF) [file pgen.1002857.s009.tif]

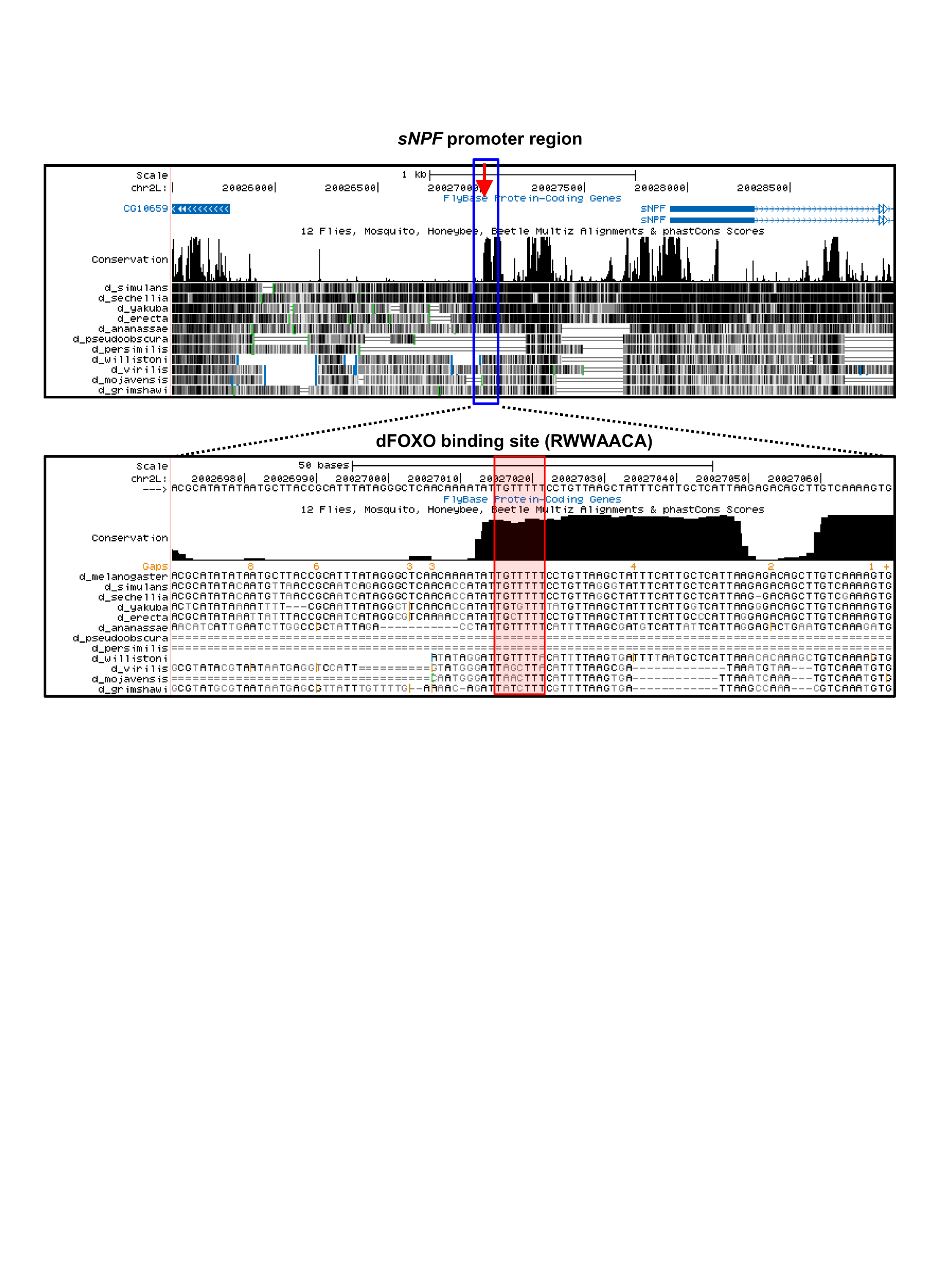

Supplement: Figure S10 — Promoter analysis of sNPF genes from twelve Drosophila species reveals that the dFOXO binding site, which is RWWAACA, was conserved in five Drosophila species including D. melanogaster (Adapted and modified from UCSC Genome Browser at http://genome.ucsc.edu). (TIF) [file pgen.1002857.s010.tif]

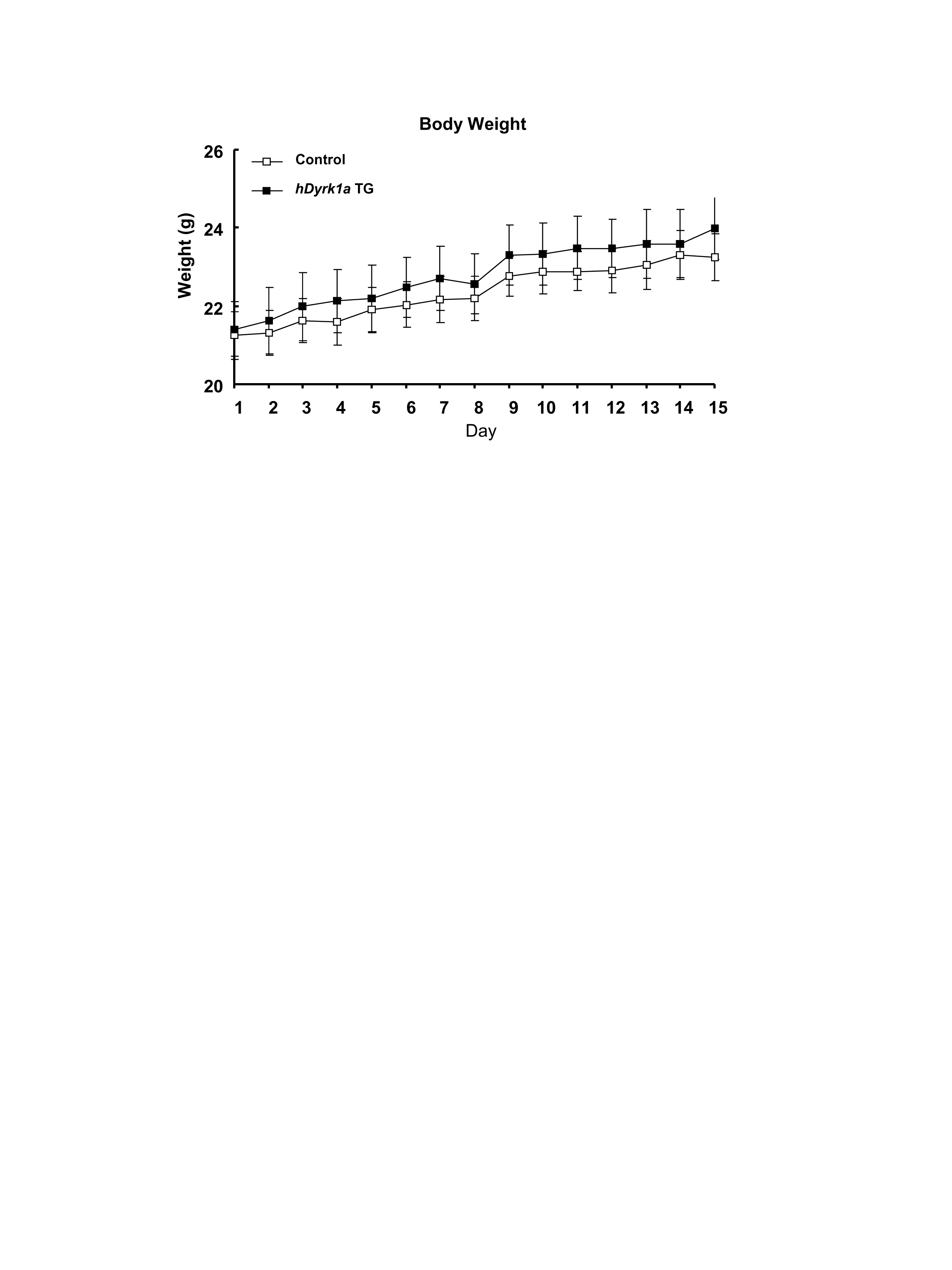

Supplement: Figure S11 — hDyrk1a transgenic mice showed slightly increased body weight. Data are presented as means ± s.e.m. *P<0.05. (TIF) [file pgen.1002857.s011.tif]
