# Supplementary material for: Minibrain/Dyrk1a Regulates Food Intake through the Sir2-FOXO-sNPF/NPY Pathway in Drosophila and Mammals
Source: PLoS Genet. 2012 Aug 2;8(8):e1002857. doi: 10.1371/journal.pgen.1002857 (PMC3410862; doi:10.1371/journal.pgen.1002857)
Supplement: Table S1 — mnb expression in the DNA microarray analysis. (DOC) [file pgen.1002857.s012.doc]

| symbol | fold change by sNPFpeptide treatment | FlyBase ID | function/biological process |
| --- | --- | --- | --- |
| minibrain | 34.1 | FBgn0259168 | protein kinase activity, olfactory learning, circadian rhythm, nervous system development, brain development. |
| CG15059 | 24.6 | FBgn0030905 | unknown |
| CG3474 | 22.6 | FBgn0028871 | structural constituent of chitin-based cuticle |
