# Supplementary material for: Minibrain/Dyrk1a Regulates Food Intake through the Sir2-FOXO-sNPF/NPY Pathway in Drosophila and Mammals
Source: PLoS Genet. 2012 Aug 2;8(8):e1002857. doi: 10.1371/journal.pgen.1002857 (PMC3410862; doi:10.1371/journal.pgen.1002857)
Supplement: Table S2 — PCR primer sequences in this study. (DOC) [file pgen.1002857.s013.doc]

| gene | product size | PCR primer sequences | Usage |
| --- | --- | --- | --- |
| *mnb* | 204 bp | forward : CGTGACTTCAATTGGATAAATAGCT  reverse : GCTGAAATTGACCCTCAGGTCC | RT-PCR |
| *Dyrk1a** | 115 bp | forward : GGGAACGAGTAACAGTGGGA  reverse : ACCTGGGGACTGTGTGTCTC | RT-PCR |
| *sNPF* | 188 bp | forward : CCCGAAAACTTTTAGACTCA  reverse : TTTTCAAACATTTCCATCGT | RT-PCR |
| *Dilp2* | 183 bp | forward : GTATGGTGTGCGAGGAGTAT  reverse : TGAGTACACCCCCAAGATAG | RT-PCR |
| *Dilp3* | 216 bp | forward : AAGCTCTGTGTGTATGGCTT  reverse : AGCACAATATCTCAGCACCT | RT-PCR |
| *Dilp5* | 211 bp | forward : AGTTCTCCTGTTCCTGATCC  reverse : CAGTGAGTTCATGTGGTGAG | RT-PCR |
| *NPY** | 76bp | forward : CTCCGCTCTGCGACACTACA  reverse : AATCAGTGTCTCAGGGCTGG | RT-PCR |
| *rp49* | 122 bp | forward : AGGGTATCGACAACAGAGTG  reverse : CACCAGGAACTTCTTGAATC | RT-PCR |
| *GAPDH** | 199 bp | forward : GTATTGGGCGCCTGGTCACC  reverse : CGCTCCTGGAAGATGGTGATGG | RT-PCR |
| *Actin5C* | 176 bp | forward : GTGCCCATCTACGAGGGTTA  reverse : AGGGCAACATAGCACAGCTT | ChIP-PCR |
| *Mnb-CRE* | 141 bp | forward : CTGAGGTCTGGGTTTTACCG  reverse : AGACAGACAGCCAAGCCAAG | ChIP-PCR |

*** mouse gene
